# CB_1_R and iNOS are distinct players promoting pulmonary fibrosis in Hermansky–Pudlak syndrome

**DOI:** 10.1002/ctm2.471

**Published:** 2021-07-04

**Authors:** Resat Cinar, Joshua K. Park, Charles N. Zawatsky, Nathan J. Coffey, Steven P. Bodine, Jasmina Abdalla, Tadafumi Yokoyama, Tony Jourdan, Lindsey Jay, Mei Xing G. Zuo, Kevin J. O'Brien, Junfeng Huang, Ken Mackie, Asaf Alimardanov, Malliga R. Iyer, William A. Gahl, George Kunos, Bernadette R. Gochuico, May Christine V. Malicdan

**Affiliations:** ^1^ Section on Fibrotic Disorders National Institute on Alcohol Abuse and Alcoholism, National Institutes of Health Maryland USA; ^2^ Laboratory of Physiologic Studies National Institute on Alcohol Abuse and Alcoholism National Institutes of Health Rockville Maryland USA; ^3^ Section of Human Biochemical Genetics Medical Genetics Branch National Human Genome Research Institute National Institutes of Health Bethesda Maryland USA; ^4^ Therapeutics Development Branch Division of Preclinical Innovation National Center for Advancing Translational Sciences National Institutes of Health Rockville Maryland USA; ^5^ Department of Psychological and Brain Sciences Indiana University Bloomington Indiana USA; ^6^ NIH Undiagnosed Diseases Program and Office of the Clinical Director National Human Genome Research Institute National Institutes of Health Bethesda Maryland USA; ^7^ Present address: Department of Pediatrics Kanazawa University Kanazawa Japan; ^8^ Present address: INSERM Lipids, Nutrition, Cancer UMR1231 University of Burgundy and Franche‐Comté Dijon France

**Keywords:** endocannabinoids, fibrosis, lung disease, polypharmacology, rare disease

## Abstract

Hermansky–Pudlak syndrome (HPS) is a rare genetic disorder which, in its most common and severe form, HPS‐1, leads to fatal adult‐onset pulmonary fibrosis (PF) with no effective treatment. We evaluated the role of the endocannabinoid/CB_1_R system and inducible nitric oxide synthase (iNOS) for dual‐target therapeutic strategy using human bronchoalveolar lavage fluid (BALF), lung samples from patients with HPS and controls, HPS‐PF patient‐derived lung fibroblasts, and bleomycin‐induced PF in pale ear mice (HPS1^ep/ep^). We found overexpression of CB_1_R and iNOS in fibrotic lungs of HPSPF patients and bleomycin‐infused pale ear mice. The endocannabinoid anandamide was elevated in BALF and negatively correlated with pulmonary function parameters in HPSPF patients and pale ear mice with bleomycin‐induced PF. Simultaneous targeting of CB_1_R and iNOS by MRI‐1867 yielded greater antifibrotic efficacy than inhibiting either target alone by attenuating critical pathologic pathways. Moreover, MRI‐1867 treatment abrogated bleomycin‐induced increases in lung levels of the profibrotic interleukin‐11 via iNOS inhibition and reversed mitochondrial dysfunction via CB_1_R inhibition. Dual inhibition of CB_1_R and iNOS is an effective antifibrotic strategy for HPSPF.

## Graphical abstract

Overactivity of CB_1_R and iNOS contributes to HPSPF development and progression via multiple critical pathological processes including dysregulation of mitochondrial function and IL‐11. Simultaneous inhibition of lung CB_1_R and iNOS by a hybrid inhibitor (MRI‐1867) is a rational therapeutic strategy for achieving therapeutic efficacy in HPSPF with attenuating fibrosis, improving pulmonary function and survival.

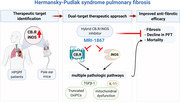

## 1 INTRODUCTION

Hermansky–Pudlak syndrome (HPS) is a rare autosomal recessive disorder of lysosome‐related organelle biogenesis caused by bi‐allelic mutations in any of 11 different genes.^1^, ^2^ HPS is reported worldwide, with an estimated prevalence of 1–9 per 1,000,000 individuals. HPS is highly prevalent in northwest Puerto Rico, with an estimated prevalence of ∼1 in 1800. To date, 1407 individuals with HPS are registered with the HPS Network (www.hpsnetwork.org; personal communication of Donna Appell, R.N.). Middle‐aged adults with HPS‐1 or HPS‐4 and children with HPS‐2 develop HPS pulmonary fibrosis (HPSPF),^3^ a leading cause of death in these patients.^4^, ^5^ HPS‐1 patients develop the most common and severe form of HPSPF. Lung histopathology of HPSPF patients resembles that of patients with idiopathic pulmonary fibrosis (IPF), with the additional features of foamy alveolar macrophages (AMs) and enlarged alveolar type II (ATII) cells.^6^, ^7^ In the absence of a currently available FDA‐approved therapy for HPSPF, there is an urgent need for identifying new therapeutic targets and treatment strategies. Pirfenidone was approved for the treatment of IPF, but its efficacy in IPF is moderate, and in HPSPF, inconclusive.^8^, ^9^

The pathogenesis of HPSPF is complex^8^ and can involve multiple factors, including alveolar inflammation, epithelial dysfunction, and fibrogenesis; therefore, simultaneously targeting multiple pathways may improve therapeutic efficacy. Indeed, HPS patients exhibit alveolar inflammation with accumulation of activated AMs that precedes fibrosis.^10^ Furthermore, epithelial cell dysfunction in HPS has been hypothesized to increase susceptibility to fibrosis due to repetitive injuries.^11^

Alveolar inflammation and epithelial dysfunction were shown to be mediated by common pathways. Inducible nitric oxide synthase (iNOS) catalyzes the generation of proinflammatory reactive nitrogen species and is involved in cell injury and apoptosis. In HPS mouse models, elevated iNOS expression contributed to hyper‐responsiveness of AMs and ATII cells and resulted in increased nitrosative stress during alveolar inflammation.^12^, ^13^ Similarly, in HPS‐1 patients, increased nitrosylation was correlated with disease severity.^12^ In IPF, iNOS is induced in fibrotic lung tissues in mice^14^, ^15^ and humans,^16^ and is thought to promote lung inflammation and fibrosis progression.^17^, ^18^, ^19^

Another emerging potential target for HPSPF could be the endocannabinoid/CB_1_R system. Endocannabinoids are lipid‐signaling molecules. Endocannabinoids acting via CB_1_R are proinflammatory and profibrotic, promoting fibrosis progression in multiple organs, including the liver,^20^, ^21^, ^22^ kidney,^23^ heart,^24^ and skin.^25^ CB_1_R was also linked to radiation‐induced PF in mice^26^ and IPF.^27^ CB_1_R‐mediated mitochondrial dysfunction and endoplasmic reticulum stress have been shown to have a prominent role in multiple pathologies, such as obesity, diabetes, and cancer.^28^, ^29^, ^30^, ^31^ Mitochondrial dysfunction induces profibrotic stimuli across different organs, including the lung,^32^ which has been linked to age‐related susceptibility to PF.^33^ Recently, CB_1_R overactivation in AMs was shown to be responsible for the activation of proinflammatory and profibrogenic macrophages.^27^ CB_1_R activation also regulates MCP‐1 and TGFβ‐1 levels in PF,^27^ two molecules which have been demonstrated to play critical roles in the onset of lung fibrosis.^34^

In this work, we show that overactivity of CB_1_R and iNOS in lung tissue contributes to the progression of PF in HPS. Targeting both iNOS and CB_1_R in HPSF mice attenuated the progression of PF in HpsPF. Our studies demonstrate that simultaneous inhibition of lung CB_1_R and iNOS is a rational strategy for achieving therapeutic efficacy in HPSPF.

## 2 RESULTS

### 2.1 Overexpression of CB_1_R and iNOS protein in fibrotic lung tissue from HPSPF patients

**FIGURE 1:**
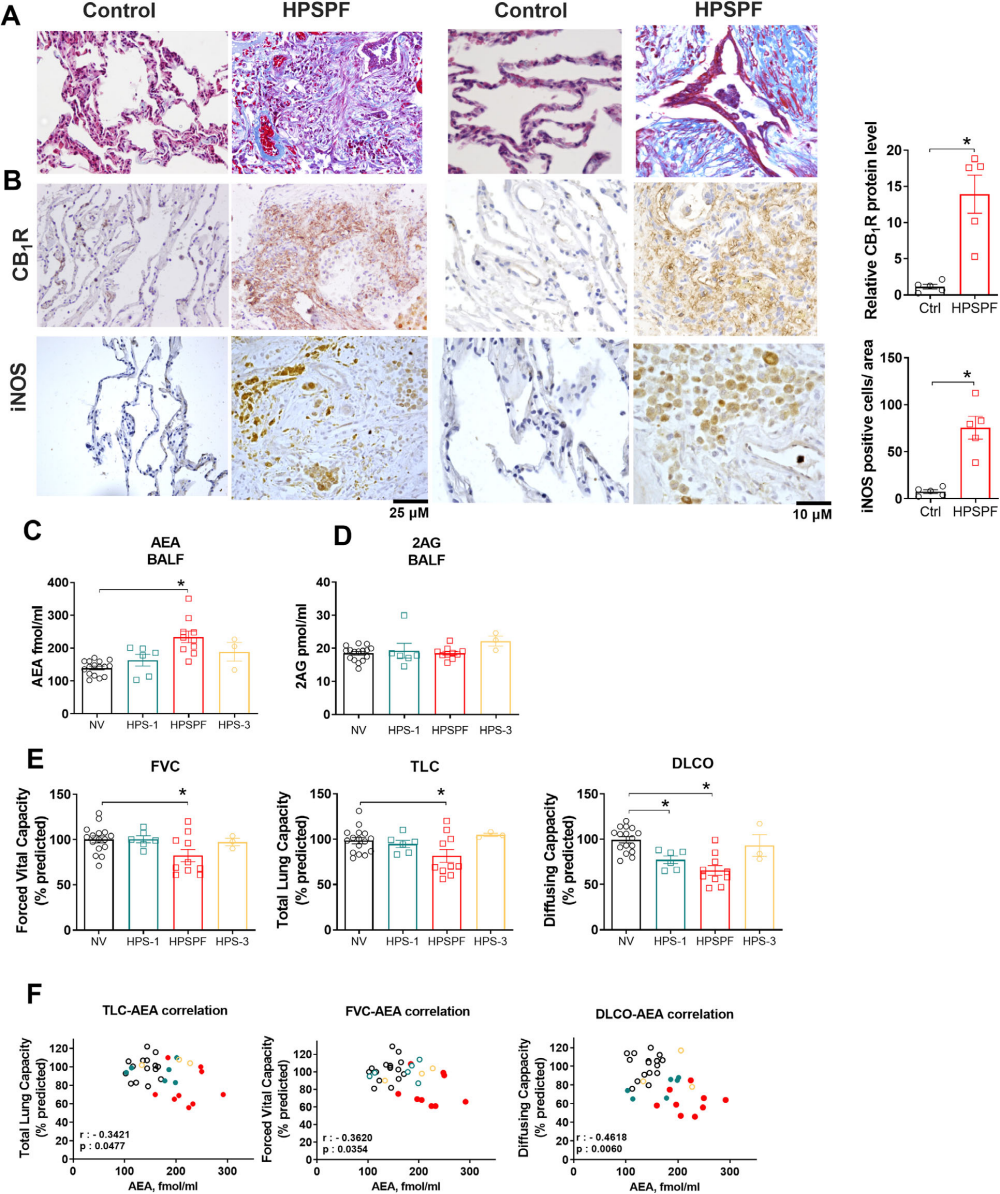
Histological and biochemical evidence from patients for pathologic involvement of endocannabinoid/CB_1_R and iNOS systems in HPSPF. Masson trichrome staining sections from HPSPF patients and controls without fibrotic lung disease (A). CB_1_R and iNOS immunohistochemistry from the same subjects (B). AEA (C) and 2‐AG (D) levels in BALF from normal volunteers (NV), HPS‐1 patients without fibrosis, patients with HPSPF and HPS‐3 patients. Pulmonary function tests (PFTs) in the same groups (E). Abbreviations: DLCO, diffusion capacity; FVC, forced vital capacity; TLC, total lung capacity. Correlation between PFTs and AEA in BALF in NV, HPS‐1, and HPSPF (F). NV: empty black symbol, HPS1: blue symbol, HPSPF: red filled symbol, HPS3: empty yellow symbol. Correlation was calculated by using Pearson correlation coefficients. Data represent mean ± SEM from five subjects for histology in each group, and 16 NV, 6 HPS‐1, 10 HPSPF, and 3 HPS‐3 subjects for BALF and PFTs. Data were analyzed by *t*‐test for comparison of histological scoring and by one‐way ANOVA followed by Dunnett's multiple comparisons test for endocannabinoids and PFT. * (*p *< 0.05) indicates significant difference from the NV group

We evaluated CB_1_R and iNOS protein levels by immunohistochemistry in lung tissue samples from control without fibrotic lung disease and HPSPF patients (Figure 1A). Both CB_1_R and iNOS proteins were dramatically increased in HPSPF lung (Figure 1B).

### 2.2 Increased anandamide levels in bronchoalveolar lavage fluid from HPSPF patients are negatively correlated with pulmonary function tests

We have recently reported that AEA, an endogenous agonist of CB_1_R receptors, is increased in bronchoalveolar lavage fluid (BALF) of IPF patients.^27^ Prompted by the hypothesis that overactivity of the endocannabinoid/CB_1_R system contributes to PF, we assessed the status of endocannabinoids in the BALF by measuring levels of AEA and 2‐arachidonoyl glycerol (2AG). In human BALF samples (Table 1), AEA was significantly elevated in HPSPF relative to normal volunteers, whereas there was no change in AEA level in HPS‐1 patients without PF (Figure 1C). HPS‐3 samples were used as negative controls because HPS‐3 patients do not develop PF. In contrast, 2AG levels in BALF were not altered (Figure 1D). Increased AEA levels in BALF were negatively correlated with pulmonary function test (PFT) results in these patients (Figure 1E, F). These findings suggest that high concentrations of AEA in BALF are related to fibrogenic processes in lungs.

**TABLE 1:** Human subject characteristics for BALF samples

	NV (*n* = 16)	HPSPF (*n* = 10)	HPS‐1 (*n* = 6)	HPS‐3 (*n* = 3)
Age (years)	35.69 ± 2.80	45.1 ± 2.47	36.67 ± 2.72	30.67 ± 7.05
Gender (M/F)	12/4	4/6	4/2	2/1

Note: Data represent mean ± SEM.

Abbreviations: F, female; HPSPF, Hermansky–Pudlak syndrome pulmonary fibrosis; IPF, idiopathic pulmonary fibrosis; M, male; NV, normal volunteer.

### 2.3 Overactivity of endocannabinoid/CB_1_R system and iNOS in bleomycin‐induced PF in pale ear (Hps‐1^ep/ep^) mice

Next, we analyzed the lungs of pale ear (*Hps‐1* ^ep/ep^) mice with bleomycin‐induced PF (HpsPF), to determine whether the endocannabinoids/CB_1_R system and iNOS are both activated as seen in human HPSPF. The pale ear (*Hps1* ^ep/ep^) mouse is the most widely used model of HPS‐1, as it mimics structural abnormalities of AM and ATII cells observed in patients with HPS and the development of PF can be induced with much lower doses of intratracheal bleomycin instillation than in wild‐type (wt) mice.^35^ In this study, PF was induced in pale ear mice at 12–16 weeks of age by subcutaneous bleomycin (60 U/kg/day) delivery (HpsPF) via osmotic pump for 7 days.

**FIGURE 2:**
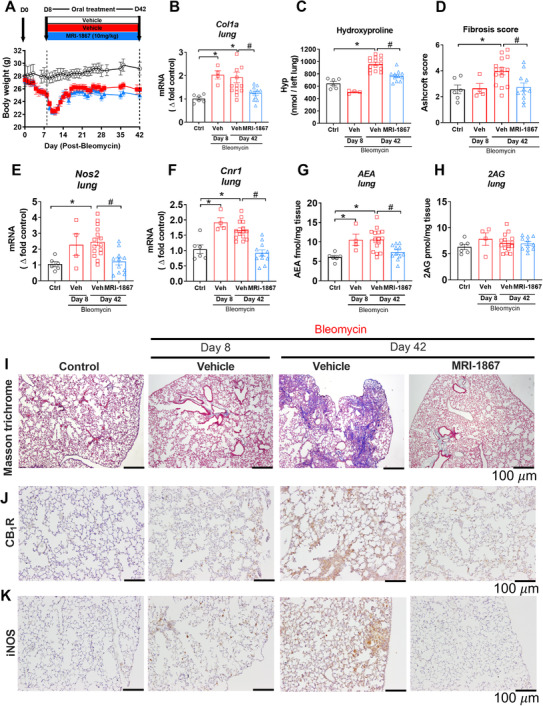
Target engagement and efficacy of MRI‐1867 in experimental model of HpsPF in pale ear mice. (A) Body weight change in Sc‐Bleo (60 U/kg)‐induced PF. (B) Gene expression of fibrosis marker collagen 1a (*Col1a*). (C) Hydroxyproline content of the left lung. (D) Ashcroft scoring from the Masson trichrome staining. Lung tissue gene expression of *Nos2* (E) and *Cnr1* (F). Levels of endocannabinoid AEA (G) and 2AG (H) in lung tissue. Masson trichrome staining (I). CB_1_R (J) and iNOS (K) immunostainings from lung tissue sections from control and bleomycin (60 U/kg) challenged pale ear mice. Data represent mean ± SEM from 6 control (Ctrl, pale ear mice infused with saline instead of bleomycin), 4 HpsPF with bleomycin+vehicle at day 8 (Veh), 15 HpsPF with bleomycin+ vehicle at day 42 (Veh), and 11 HpsPF with bleomycin+MRI‐1867 (MRI‐1867) at day 42. Data were analyzed by one‐way ANOVA followed by Dunnett's multiple comparisons test. * (*p *< 0.05) indicates significant difference from the control group. # (*p *< 0.05) indicates significant difference from the HpsPF mice treated with vehicle (Veh) at 42 day

The expression levels of *Nos2* and *Cnr1*, encoding iNOS and CB_1_R, respectively, in pale ear mice are similar to those in healthy control wt mice (Figure S1). Eight days after osmotic minipump implantation, gene expression of the fibrogenic marker *Col1a* was significantly increased in pale ear mice (Figure 2A, B), although no quantifiable fibrosis was observed biochemically (Figure 2C) or histologically (Figure 2D). Fibrosis was evident 42 days after initial bleomycin treatment (Figure 2C, D). Gene expression of *Nos2* (Figure 2E) and *Cnr1* (Figure 2F), along with gene expression of fibrogenic markers (Figure 2B), increased at 8 days post‐bleomycin and remained elevated at 42 days post‐bleomycin. In parallel with the findings in patients with HPS‐1, AEA (Figure 2G) but not 2AG (Figure 2H) was similarly increased in the lungs of HpsPF mice. These findings suggest that both CB_1_R and iNOS may be involved in fibrosis initiation and progression in the mouse model of HPS, which aligns with our observations in human HPSPF.

### 2.4 Localization of CB_1_R and iNOS in fibrotic lungs in pale ear mice

Based on the histological evidence, CB_1_R and iNOS expression are widely distributed in fibrotic lungs of both HPSPF patients (Figure 1) and in HpsPF (Figures 1 and 2). Indeed, both CB_1_R and iNOS are expressed in AT1 and AT2 epithelial cells, macrophages, pericytes, endothelial cells, and fibroblasts in fibrotic lungs of pale ear mice (Figure S2). These findings suggest that both proteins may be involved in multiple pathological processes in different cell types during PF. More importantly, these results suggest that dual‐targeting of CB_1_R and iNOS may provide improved antifibrotic efficacy compared to targeting either CB_1_R or iNOS alone.

### 2.5 Pharmacokinetics of MRI‐1867 in pale ear (Hps1^ep/ep^) mice

Since overactivation of CB_1_R and iNOS was observed in HPSPF patients and HpsPF mice specimens, we targeted CB_1_R and iNOS by a peripherally restricted hybrid CB_1_R/iNOS inhibitor (MRI‐1867)^36^ in an animal model for HPSPF.^37^, ^38^

MRI‐1867 caused maximal inhibition of both CB_1_R and iNOS in lungs at 10 mg/kg dose in bleomycin‐induced pulmonary fibrosis in wt mice, which attenuated PF.^27^ We first assessed the pharmacokinetics and lung exposure of MRI‐1867 in pale ear and wt (C57BL/J) mice. MRI‐1867 was administered by oral gavage at 10 mg/kg. Peak plasma concentration (*C*_max_) was reached at 2 h in both wt and pale ear mice (Figure S3A). However, *C*_max_ and the area under the curve (AUC) were less than half for pale ear mice compared to wt mice for a 24‐h period (Figure S3A). Although systemic exposure was lower in pale ear mice (Figure S3A), lung exposure at the *T*_max_, 2 h after oral dosing was slightly higher in pale ear mice (25 *μ*M) compared to wt mice (20 *μ*M) (Figure S3B).

From our recent study, we are aware that bleomycin induces drug efflux transporters in murine lung and, therefore, reduces lung exposure to pharmaceutical drugs that are substrates for efflux transporters.^39^ Therefore, we tested lung exposure of MRI‐1867 in a bleomycin‐induced PF model using two different delivery routes—either oropharyngeal instillation (OP‐bleo) (0.3 U/kg) or subcutaneous osmotic minipump (SC‐bleo) (60 U/kg/day) for 7 days in pale ear mice. Both delivery routes at the selected doses generate similar, extensive PF as quantified by lung hydroxyproline content (Hyp), a biochemical marker of fibrosis (Figure S4A, B). Systemic exposure to MRI‐1867 was similar in both OP‐bleo and SC‐bleo compared to control, unchallenged pale ear mice (Figure S4C, D). However, lung exposure to MRI‐1867 was significantly diminished to 2 *μ*M in the OP‐bleo model (Figure S4E), whereas it was 30 *μ*M in the SC‐bleo model (Figure S4F), and comparable to control. This suggests that the use of a subcutaneous osmotic minipump (60 U/kg/day) in pale ear mice provides optimal delivery of bleomycin to induce PF while retaining MRI‐1867 target organ exposure.

### 2.6 Dual‐inhibition of CB_1_R and iNOS by MRI‐1867 attenuated fibrosis progression in bleomycin‐induced PF in pale ear (Hps‐1^ep/ep^) mice

Since both the endocannabinoid/CB_1_R system and iNOS could contribute to PF initiation and progression (Figure 2), we began pharmacological treatment at the fibrosis initiation phase for antifibrotic efficacy testing (Figure 2A). Single daily oral administration of MRI‐1867 from day 8 to day 42 completely abrogated increased gene expression of *Nos2* (Figure 2E) and *Cnr1* (Figure 2F), tissue level of AEA (Figure 2G), and protein expression of CB_1_R (Figure 2J) and iNOS (Figure 2K) in the lungs of HpsPF mice. This demonstrates target engagement by MRI‐1867 of both CB_1_R and iNOS in the lungs in bleo‐induced PF. Accordingly, MRI‐1867 administered orally significantly attenuated PF progression in HpsPF mice as monitored biochemically (Figure 2C) and histologically (Figure 2D, I).

### 2.7 MRI‐1867 prevents bleomycin‐induced decline in pulmonary function in pale ear mice

**FIGURE 3:**
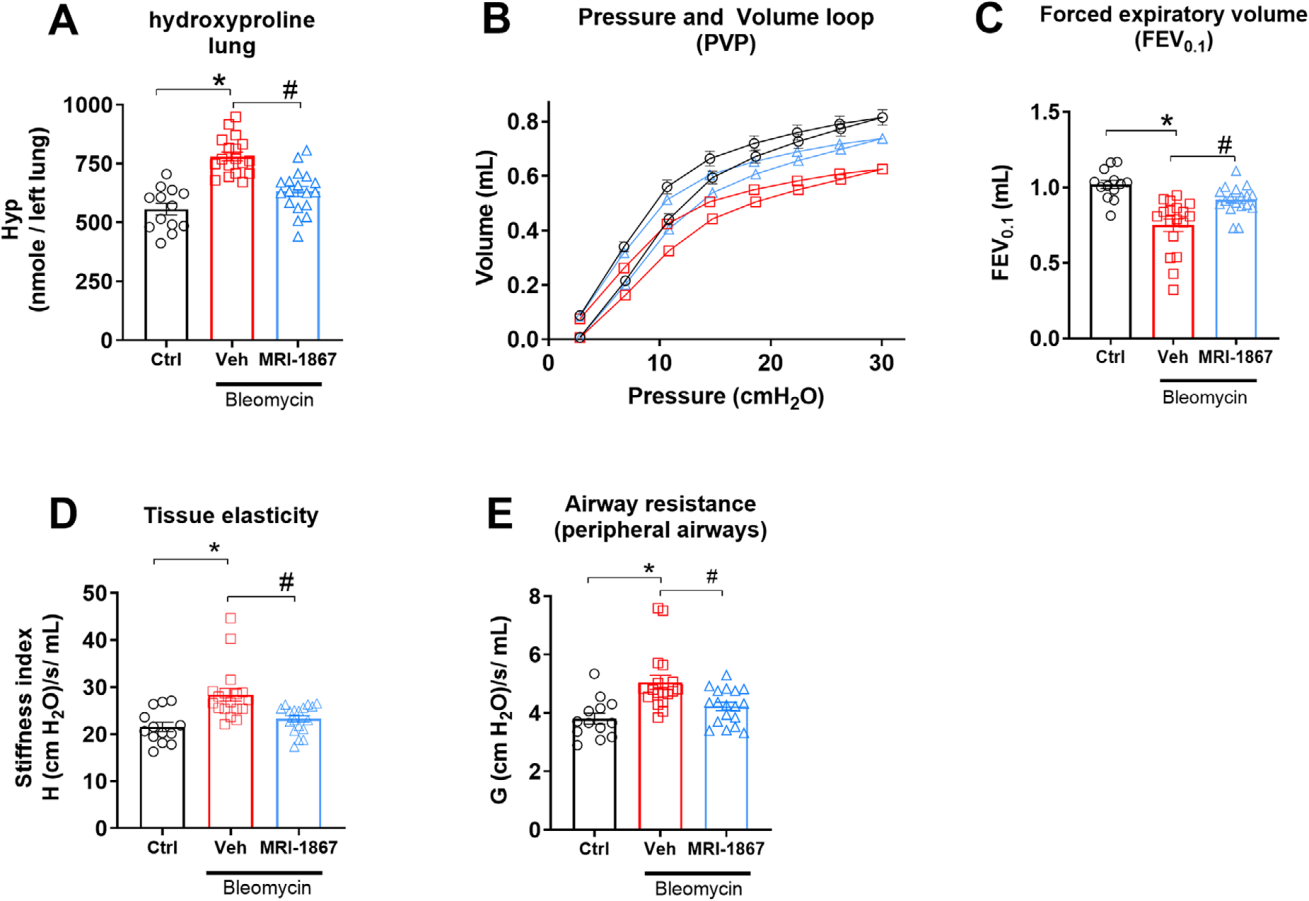
Dual target inhibition of CB_1_R and iNOS prevented decline with PF in HpsPF mice. (A) Hydroxyproline content as fibrosis measurement. (B) Pressure‐volume curve, (C) forced expiratory volume, (D) tissue elasticity, and (E) tissue damping as measures of lung function. Data represent mean ± SEM. *n* = 13 mice for control (Ctrl, pale ear mice infused with saline instead of bleomycin), *n* = 18 HpsPF mice for vehicle (Veh), and *n* = 18 HpsPF mice for MRI‐1867 (MRI‐1867)‐treated groups. Data were analyzed by one‐way ANOVA followed by Dunnett's multiple comparisons test. * (*p *< 0.05) indicates significant difference from the control group. # (*p *< 0.05) indicates significant difference from the HpsPF mice treated with vehicle (Veh) at 42 day

PFT is a widely used clinical parameter for monitoring disease progression in PF. Therefore, we conducted PFT in a separate cohort of pale ear mice to further test the therapeutic potential of MRI‐1867 using clinically relevant physiologic outcome measures (Figure 3). At 42 days post‐bleo, in addition to attenuating fibrosis (Figure 3A), MRI‐1867 treatment significantly mitigated adverse changes in pulmonary function parameters, including lung compliance (pressure‐volume [PV] loops) (Figure 3B), airflow (forced expiratory volume [FEV] at 0.1 s) (Figure 3C), stiffness (tissue elasticity) (Figure 3D), and airway resistance (tissue damping) (Figure 3E).

### 2.8 Survival rate is significantly increased by dual inhibition of peripheral CB_1_R and iNOS in pale ear mice with bleomycin‐induced PF

Severe and progressively restrictive lung disease associated with reduced pulmonary function is a significant cause of mortality in HPSPF. We did not observe significant mortality with the SC‐bleo model using 12‐ to 16‐week‐old mice with average body weights of 24–28 g (Figures 2 and 3). However, significant mortality was evident in 24‐ to 26‐week‐old mice with average weights of 33 g (Figure S5). Osmotic minipumps deliver a systemic dose proportional to body weight, thus total bleomycin amount was higher in older mice due to their higher body weight, which may have led to increased mortality. Therefore, we used 24–26 weeks old mice as a cohort for testing the efficacy of MRI‐1867 on survival (Figure S5A, B). MRI‐1867 significantly increased survival rate at 5 weeks to 87.5% compared to 50% in the vehicle group (Figure S5B) and prevented the decline in PFT parameters (Figure S5C) in pale ear mice.

### 2.9 Increased BALF levels of endocannabinoids reversed by CB_1_R antagonism in pale ear mice with PF

**FIGURE 4:**
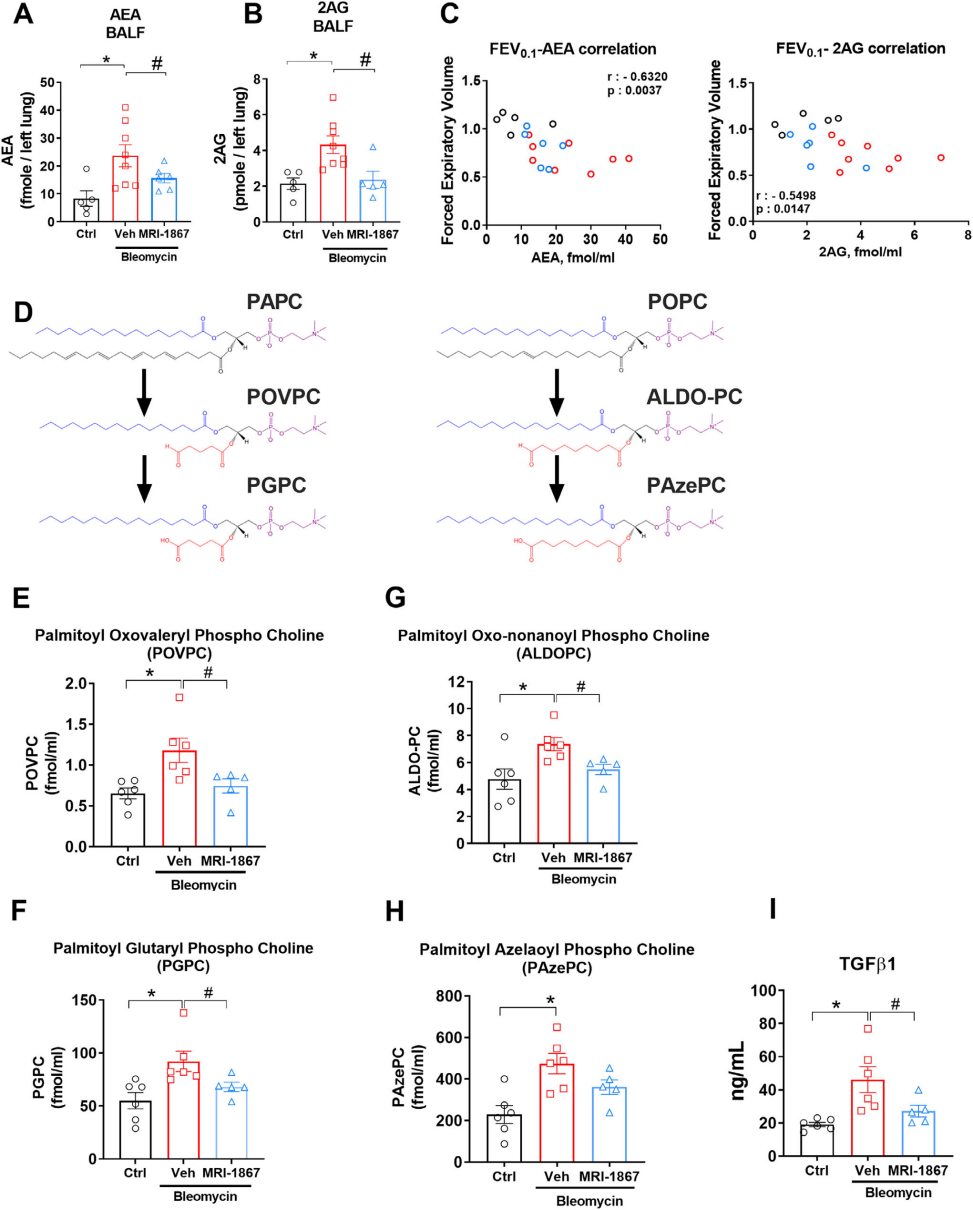
Bleomycin‐induced elevation of endocannabinoids, truncated oxidized phospholipids, and TGFβ‐1 in BALF attenuated by MRI‐1867 treatment. Levels of AEA (A) and 2AG (B) in BALF of HpsPF mice and control. Correlation with forced expiratory volume 0.1 s (FEV_0.1_) as pulmonary function tests (PFTs) and endocannabinoids (C) in BALF. (D) Chemical structures of surfactant phosphatidyl cholines PAPC and POPC and their truncated oxidized phosphatidyl cholines (POVPC, PGPC, ALDOPC, and PAzePC). Levels of POVPC (E), PGPC (F), ALDOPC (G), PAzePC (H), and TGFβ‐1 (I) in BALF of pale ear mice. Data represent mean ± SEM. *n* = 6 pale ear mice for control (Ctrl, pale ear mice infused with saline instead of bleomycin), *n* = 8 HpsPF mice for vehicle (Veh), and *n* = 6 HpsPF mice for MRI‐1867 (MRI‐1867)‐treated groups for endocannabinoid measurements. *n* = 6 HpsPF mice for control (Ctrl), *n* = 6 HpsPF mice for vehicle (Veh), and *n* = 5 HpsPF mice for MRI‐1867 (MRI‐1867)‐treated groups for oxidized phospholipids and TGFβ‐1 measurements. Data were analyzed by one‐way ANOVA followed by Dunnett's multiple comparisons test. * (*p *< 0.05) indicates significant difference from the control group. # (*p *< 0.05) indicates significant difference from the HpsPF mice treated with vehicle (Veh) at 42 day

The increase in AEA BALF from HPSPF patients and its negative correlation with PFTs (Figure 1F) suggested its pathogenic role in HPSPF (Figure 1C). AEA is one of several endogenous agonists for CB_1_R. Since dual inhibition of CB_1_R and iNOS prevented a decline in PFT parameters (Figure 3), we measured endocannabinoid levels in BALF in pale ear mice. Indeed, both AEA (Figure 4A) and 2AG (Figure 4B) levels were elevated in BALF of bleomycin‐challenged pale ear mice. This increase in AEA and 2AG was completely abolished by MRI‐1867 treatment (Figure 4A, B). In accordance with our findings in HPS patients (Figure 1F), AEA levels in BALF negatively correlated with FEV_0.1_ in HpsPF mice (Figure 4C).

### 2.10 Increased levels of truncated oxidized phosphatidylcholines and TGFβ‐1 in BALF are diminished by MRI‐1867 treatment in pale ear mice

One of the distinct features of HPS is the presence of foamy AMs in the lungs. There is evidence from experimental PF studies in wild‐type mice that foamy cell formation in AM contributes to PF development by lipid dysregulation via an AT2 cell‐macrophage paracrine lipid axis.^40^ Notably, truncated oxidized phosphatidylcholines (OxiPCs) have been deemed responsible for inducing profibrogenic M2 macrophage polarization, TGF‐β1 production, foamy cell formation, and fibrogenesis.^40^ Additionally, we recently showed that CB_1_R activation increased truncated OxiPCs during acute lung injury and CB_1_R antagonism completely prevented OxiPCs generation and lung injury.^41^ Therefore, we explored the status of OxiPCs, such as palmitoyl oxovaleryl phosphatidyl choline (POVPC) and palmitoyl glutaryl phosphatidyl choline (PGPC), as well as palmitoyl oxo‐nonanoyl phosphatidyl choline (ALDOPC) and palmitoyl azelaoyl phosphatidyl choline (PAzePC), which are, respectively, the truncated oxidized products of surfactant phospholipids palmitoyl arachidonyl phosphatidyl choline (PAPC) and palmitoyl oleoyl phosphatidyl choline (POPC), in the BALF of pale ear mice with bleomycin‐induced PF (Figure 4D). Bleomycin increased the levels of POVPC (Figure 4E), PGPC (Figure 4F), ALDOPC (Figure 4G), and PAzePC (Figure 4H) in HpsPF BALF, while MRI‐1867 treatment significantly attenuated these increases. Additionally, bleo‐induced increases in TGF‐β1 in BALF were also completely attenuated by MRI‐1867 treatment (Figure 4I).

### 2.11 Simultaneous dual inhibition of CB_1_R and iNOS is more effective in attenuating HpsPF than the single targeting of CB_1_R or iNOS alone

**FIGURE 5:**
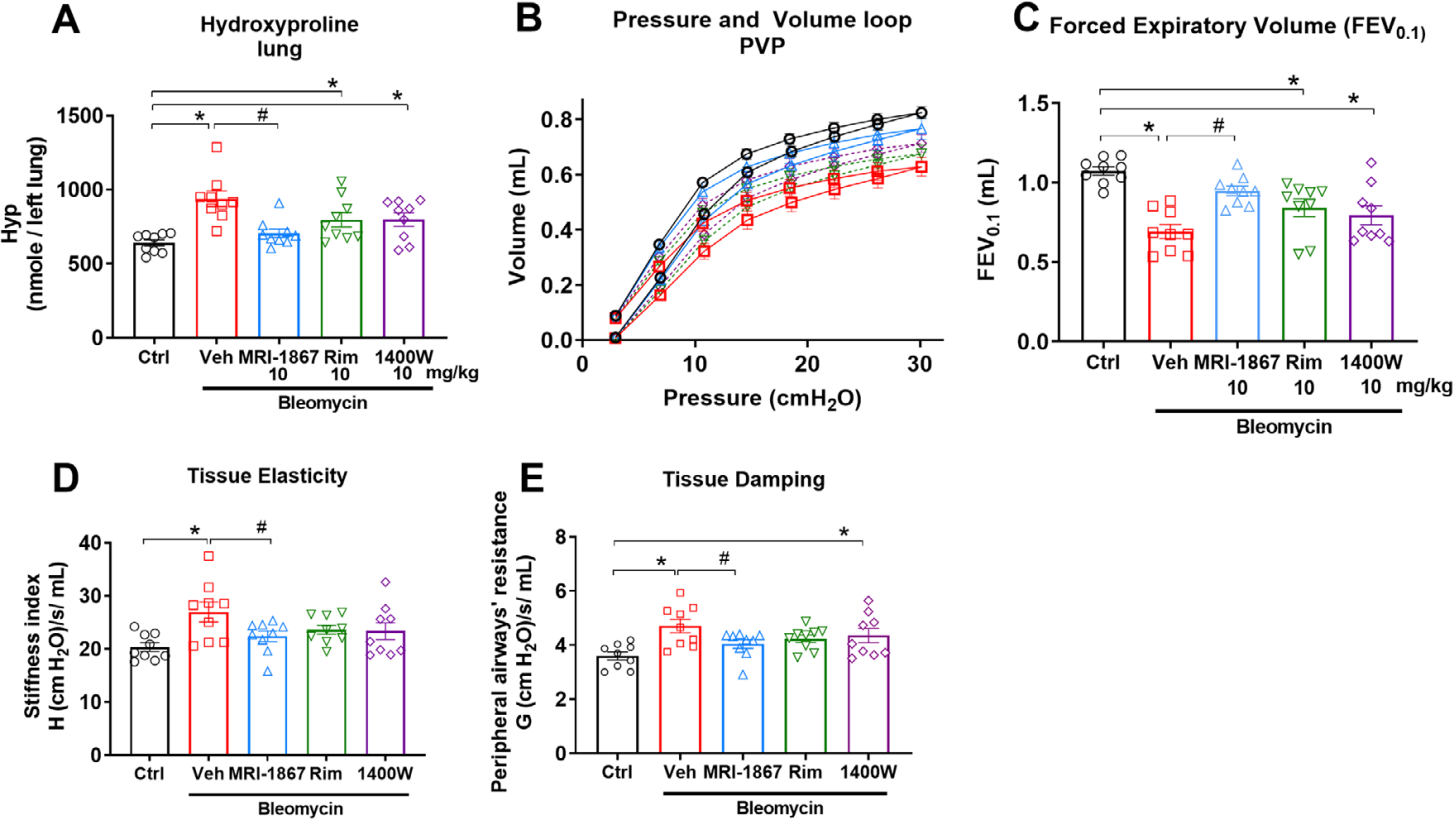
Only MRI‐1867, but not rimonabant or 1400W, reduced fibrosis and improved PFT in pulmonary fibrosis in HpsPF mice. (A) Hydroxyproline content as fibrosis measurement. (B) Pressure‐volume curve, (C) forced expiratory volume, (D) tissue elasticity, and (E) tissue damping as measures of lung function. Data represent mean ± SEM. *n* = 9 mice per group. Control group (Ctrl) were pale ear mice infused with saline instead of bleomycin. HpsPF mice (pale ear mice challenged with bleomycin) were either given vehicle (veh), MRI‐1867, rimonabant (Rim), or iNOS inhibitor (1400W). Data were analyzed by one‐way ANOVA followed by Dunnett's multiple comparisons test. * (*p *< 0.05) indicates significant difference from the control group. # (*p *< 0.05) indicates significant difference from HpsPF mice treated with vehicle (Veh) at 42 day

Since MRI‐1867 provided significant antifibrotic efficacy, we explored the distinct contributions of CB_1_R and iNOS to HPSPF pathology. Bleomycin‐challenged pale ear mice were treated daily between day 8 and 42 by oral gavage of vehicle, rimonabant (CB_1_R antagonist), 1400W (iNOS inhibitor), or MRI‐1867 (hybrid CB_1_R/iNOS inhibitor) at 10 mg/kg/day. Only MRI‐1867 treatment significantly attenuated PF progression in HpsPF mice (Figure 5A) and prevented declines in pulmonary function (Figure 5B–E). CB_1_R antagonism or iNOS inhibition alone caused partial improvements in bleomycin‐induced fibrosis (Figure 5A) and diminished pulmonary function (Figure 5B–E), which did not reach statistical significance. These findings underscore the value of a multitargeted approach for effective antifibrotic therapy in HPSPF.

### 2.12 MRI‐1867 treatment reversed bleomycin‐induced mitochondrial dysfunction via CB_1_R and iNOS inhibition in lungs of pale ear mice

Evidence suggests that mitochondrial dysfunction in AT2 cells is associated with increased susceptibility to PF. CB_1_R overactivation was shown to affect mitochondrial function in multiple organs, such as the liver, muscle,^43^ stomach,^44^ and kidneys^45^; however, its effect on mitochondrial biogenesis in the lungs has not been explored. We observed that CB_1_R expression was increased in AT2 cells in fibrotic lungs in pale ear mice (Figure S6A). To investigate mitochondrial dysfunction in our HpsPF model, we measured the expression of peroxisome proliferator activated receptor gamma coactivator 1 alpha (PGC1*α*), reduction of which was associated with insufficient mitochondrial biogenesis.^42^ We also determined the levels of phosphatase and TENsion homolog (PTEN)‐induced kinase 1 (PINK1), which were shown to be decreased in AT2 cells from fibrotic lungs associated with mitochondrial dysfunction and increased activation of TGF‐β1.^46^ Our results show that both PGC1*α* and PINK1 in HpsPF mice were significantly reduced at day 8, and remained low until day 42 (Figure S6B, C), indicating a significant increase in mitochondrial dysfunction. The reduction of PGC1*α* was significantly reversed by either CB_1_R antagonist (rimonabant) or hybrid CB_1_R/iNOS inhibitor (MRI‐1867), but not by an iNOS inhibitor (1400W) (Figure S6B). On the other hand, CB_1_R or iNOS inhibition alone significantly attenuated the effect of bleomycin in reducing PINK1 expression, whereas MRI‐1867 completely normalized it, suggesting the involvement of both CB_1_R and iNOS inhibition (Figure S6C). This demonstrates that CB_1_R and iNOS activation independently contribute to mitochondrial dysfunction in HPSPF, and that combined inhibition of iNOS and CB_1_R normalizes mitochondrial biogenesis markers. Furthermore, CB_1_R antagonism by either MRI‐1867 or rimonabant fully attenuated bleomycin‐induced elevation of TGFβ‐1 protein in BALF from pale ear mice (Figure S6D).

### 2.13 MRI‐1867 treatment abrogated bleomycin‐induced increase in interleukin 11 levels in the lungs via iNOS inhibition

**FIGURE 6:**
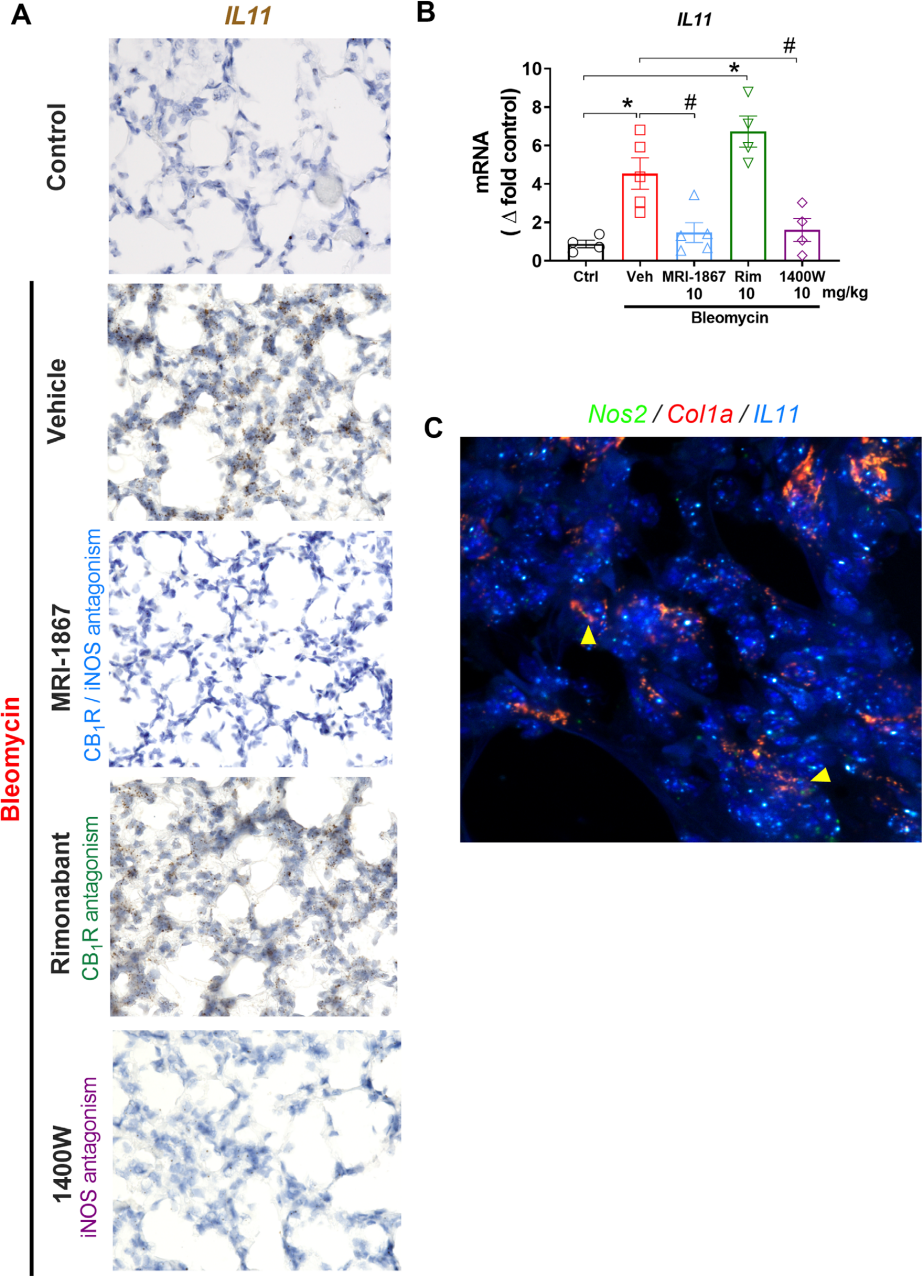
iNOS activation regulates interleukin‐11 (IL‐11) expression in lungs during bleomycin‐induced HPSPF in pale ear mice. Gene expression of IL‐11 in lung tissue sections by RNAscope *in situ* hybridization (ISH) was performed on fixed lung tissue sections (A) and by real‐time PCR in lung homogenate (B) in pale ear mice. Colocalization of Nos2 and IL11 in Col1a expressing cells by RNAscope *in situ* hybridization (ISH) (C). Data represent mean ± SEM. *n* = 4 mice for control (Ctrl, pale ear mice infused with saline instead of bleomycin), *n* = 5 HpsPF mice for vehicle (Veh), *n* = 5 HpsPF mice for MRI‐1867 (Hybrid CB_1_R/iNOS inhibitor)‐treated group, *n* = 4 HpsPF mice for rimonabant (Rim, CB_1_R antagonist), and *n* = 4 mice for 1400W (iNOS inhibitor)‐treated group. Data were analyzed by one‐way ANOVA followed by Dunnett's multiple comparisons test. * (*p *< 0.05) indicates significant difference from the control group. # (*p *< 0.05) indicates significant difference from the HpsPF mice treated with vehicle (Veh) at 42 day

Recently, interleukin 11 (IL‐11) was identified as a therapeutic target for PF because it contributes to fibroblast proliferation and promotes fibrosis,^47^ and was shown to be critical in the development of HPSPF in pluripotent cell‐derived organoids.^48^ Therefore,we speculated that IL11 expression could be increased in our HpsPF model. Indeed, we found out that bleomycin increased *IL11* gene expression level in fibrotic lungs as shown by fluorescence *in situ* hybridization (Figure 6A) and real‐time PCR (Figure 6B). The role of iNOS and CB_1_R in activating IL‐11 has not been investigated previously. Surprisingly, IL‐11 elevation was completely abrogated by either the iNOS inhibitor (1400W) or the hybrid CB_1_R/iNOS inhibitor (MRI‐1867) but not by the CB_1_R antagonist (rimonabant). This suggests that iNOS activation is responsible for inducing IL‐11 expression in PF. The expression of iNOS in Col1A positive cells in fibrotic lung (Figure 6C) could also support this hypothesis.

### 2.14 TGFβ‐1 induced activation of iNOS and endocannabinoid/CB_1_R system regulates activation of human HPSPF primary lung fibroblasts and iNOS inhibition attenuates IL‐11 expression and secretion

**FIGURE 7:**
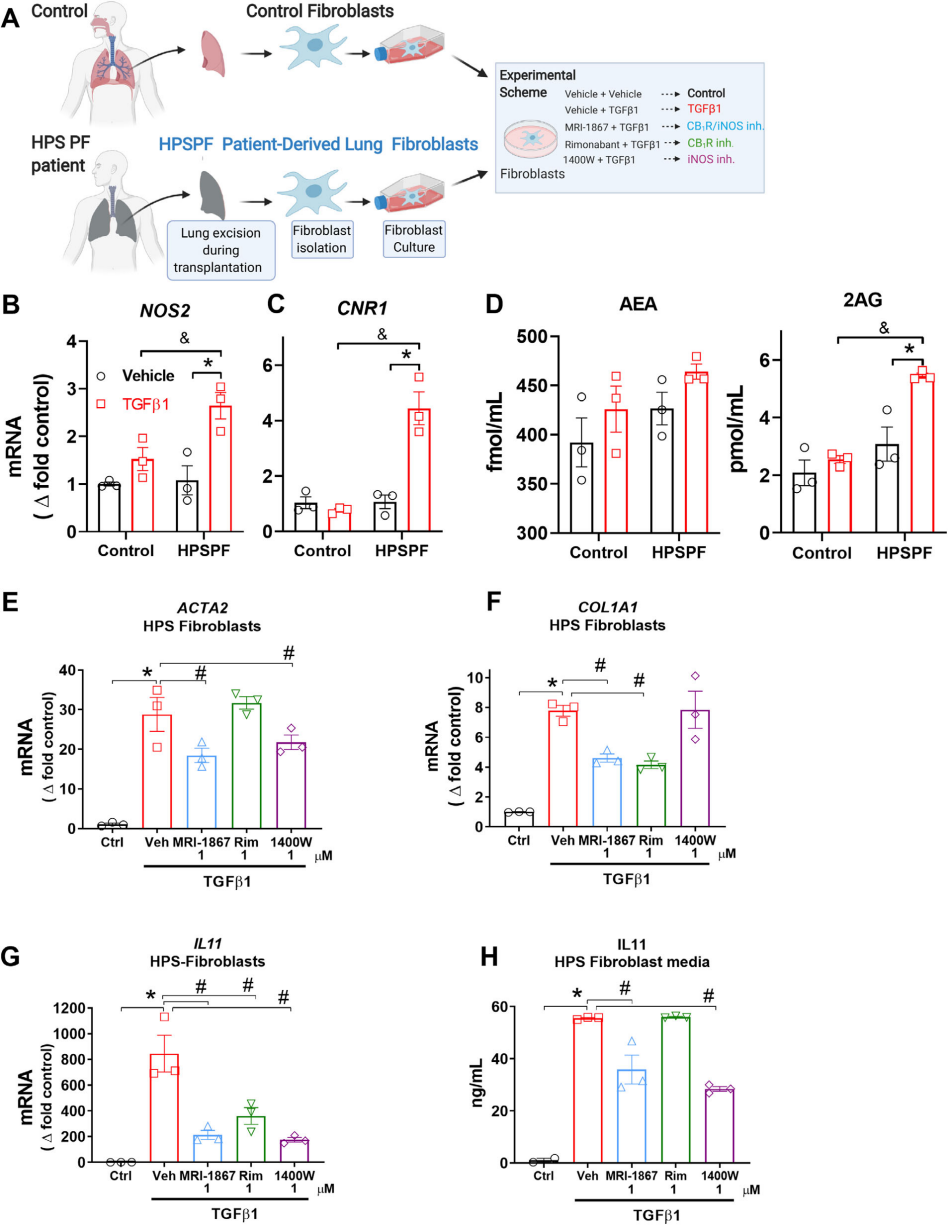
MRI‐1867 treatment reduces fibroblast activation and IL‐11 secretion via iNOS inhibition in primary lung fibroblasts derived from HPSPF patients. (A) Schematic presentation of primary fibroblast isolation and *in vitro* experimental conditions. Gene expression levels of (B) *NOS2* and (C) *CNR1* in control and HPSPF human lung fibroblasts, and (D) levels of endocannabinoids in the culture medium at 24 h after incubation in the absence or presence of TGFβ‐1 (100 ng/ml). Gene expressions of (E) *ACTA2*, (F) *COL1A1*, and (G) *IL11* in fibroblasts, and (H) IL‐11 level in culture media at 24 h after treatment with MRI‐1867, rimonabant, and 1400W in the presence of TGFβ‐1 (100 ng/ml). Data represent mean ± SEM. *n* = 3 per group. Data were analyzed by one‐way ANOVA followed by Dunnett's multiple comparisons test. * (*p *< 0.05) indicates significant difference from the control group. # (*p *< 0.05) indicates significant difference from the TGFβ‐1+ vehicle group

We further explored the regulation of IL‐11 by iNOS studying HPSPF patient‐derived primary lung fibroblasts (Figure 7) and lung fibroblasts from controls unaffected by lung disease. TGFβ‐1 induced increased gene expression of iNOS (Figure 7B), CB_1_R (Figure 7C), and the endocannabinoid, 2AG (Figure 7D) in HPSPF lung fibroblasts, but not in control cells (Figure 7B–D), indicating activation of both endocannabinoid/CB_1_R and iNOS in effector cells of human HPSPF. This observation aligns with our histological finding that both CB_1_R and iNOS proteins are highly expressed in human HPSPF lungs but not in control lungs (Figure 1B). TGFβ‐1 induced *ACTA2* and *COLA1* expression as expected due to fibroblast activation (Figure 7E, F). Pretreatment with either MRI‐1867 or 1400W, but not rimonabant, attenuated TGFβ‐1‐induced *ACTA2* expression, suggesting that iNOS contributes to fibroblast/myofibroblast activation (Figure 7E). In contrast, pretreatment with MRI‐1867 or rimonabant, but not 1400W, attenuated TGFβ‐1‐induced *COLA1* expression in HPSPF lung fibroblasts, which suggests that CB_1_R mediates collagen expression in these cells (Figure 7F). Furthermore, TGFβ‐1 significantly increased *IL11* gene expression (Figure 7H) and its secretion by fibroblasts (Figure 7I). Rimonabant pretreatment reduced gene expression of *IL‐11* (Figure 7G), but had no effect on IL‐11 secretion (Figure 7I), whereas MRI‐1867 or 1400W treatments of HPSPF lung fibroblasts significantly attenuated TGFβ‐1‐induced *IL11* gene expression (Figure 7G) and secretion (Figure 7I). Taken together, our data uncover a novel antifibrotic role of iNOS inhibition via downregulating IL‐11 in lung fibroblasts and demonstrate that inhibition of IL‐11‐mediated fibrosis is a mechanism of action of MRI‐1867.

## 3 DISCUSSION

Here, we presented preclinical evidence and clinical observations that dual‐targeting of CB_1_R and iNOS using a hybrid inhibitor is an effective antifibrotic therapeutic strategy for HPSPF. Both *in vitro* and *in vivo* data support the overactivity of the endocannabinoid/CB_1_R system and iNOS in a murine model of HpsPF and human HPSPF. The data also reveal a deleterious role of these two targets (CB_1_R and iNOS) in the pathogenesis of HPSPF. The novel hybrid drug simultaneously targets CB_1_R and iNOS to attenuate critical fibrogenic pathways in HPSPF, and its mechanism of action involves inhibition of TGF‐β1, mitochondrial dysfunction, and IL‐11.

For rare diseases like HPSPF, the translational value of preclinical experimental models is essential for finding safe and effective therapies. With limited access to patient samples, appropriate animal models facilitate our understanding of pathological pathways and the development of potential treatments. First, we found the widespread protein expression of CB_1_R and iNOS in human HPSPF lung explants, which is compatible with the potential pathogenic role of both proteins even in late stages of PF. Additionally, elevated BALF levels of the endocannabinoid anandamide (AEA) in HPSPF patients were negatively correlated with PFT results, further suggesting a pathological role of CB_1_R. However, lack of access to human lung tissue from early disease stages limited our ability to dissect the role of these proteins in the initiation and early progression of PF in HPS. Pale ear mice do not develop spontaneous PF,^38^ which requires a fibrosis inducer such as bleomycin. Although bleomycin‐induced PF model has some limitations,^39^ it is a widely used model for the preclinical assessment of potential therapies for PF.^49^ Fortunately, bleomycin‐induced PF in pale ear mice as a mouse model of HpsPF recapitulated overactivation of both the endocannabinoid/CB_1_R system and iNOS (Figure 2) observed in HPSPF patients (Figure 1). This confirmed the translational value of this animal model for further preclinical testing. Importantly, levels of iNOS, CB_1_R, and AEA (Figure 2) were increased in bleomycin challenged pale ear mice with early and established fibrosis, which suggested their potential roles in fibrosis initiation and progression.

Both CB_1_R and iNOS show similarly low expression in lungs of control wt and pale ear mice, suggesting comparable baseline expression under healthy conditions. Since pale ear mice do not develop spontaneous fibrosis, we can not directly test whether CB_1_R and/or iNOS overactivity contributes to fibrosis development in mice in the absence of a fibrosis inducer. However, in the presence of bleomycin, both iNOS and CB_1_R expression and function are remarkably increased at the early fibrosis initiation stage and then remain elevated throughout fibrosis progression (Figure 2).^27^ Endocannabinoids, CB_1_R, and iNOS are similarly overactivated in IPF^27^ and HPSPF, indicating their requisite role in PF initiation and progression.

iNOS is known to be involved in alveolar inflammation by activating AMs and for its hyperresponsiveness in HPS, which was the rationale for us to target iNOS in HPSPF. Moreover, there is evidence that iNOS is significantly overexpressed in multiple cell types during tissue injury and fibrosis. iNOS may regulate multiple downstream pathologic pathways in different cells, but its cell‐specific regulatory roles are not well understood. For instance, an early proliferative response of human pulmonary fibroblasts to inflammatory stimuli is associated with increased iNOS gene expression.^17^ In addition, iNOS protein levels in human lung tissue are strongly correlated with the severity of PF, whereas low expression of iNOS is associated with reduced risk of death in PF.^50^ Consequently, iNOS inhibitors showed antifibrotic efficacy in rodent models of experimental fibrosis.^51^ Here, we discovered a previously unrecognized role of iNOS in regulating IL‐11 expression and secretion by activated lung fibroblasts (Figure 7). It was recently shown that IL‐11 is essential for PF development in IPF and HPSPF.^47^, ^48^ Therefore, inhibiting IL‐11 expression and its signaling pathway emerged as a therapeutic goal for HPSPF. MRI‐1867 attenuated the fibrogenesis‐associated increase in IL‐11 levels via iNOS inhibition in the lungs of HpsPF mice (Figure 6) and HPSPF human lung fibroblasts (Figure 7), which suggests that iNOS regulates IL‐11. Thus, inhibiting iNOS could be sufficient to inhibit IL‐11 in PF, and our findings validate the therapeutic potential of iNOS inhibition in HPSPF.

The therapeutic efficacy of MRI‐1867 in treating HPSPF is further demonstrated by the compound's ability to attenuate mitochondrial dysfunction. Mitochondrial dysfunction, particularly in AT2 cells, is a feature of PF susceptibility, which is observed in HPS. Impairment of mitochondrial biogenesis with reduction of PGC1a, PINK1, and fatty acid oxidation in AT2 cells could exacerbate lung injury and promote PF.^42^, ^46^, ^52^ We found that MRI‐1867 normalizes PGC1a and PINK1, markers of mitochondrial biogenesis in the lung (Figure S6). Overactivation of CB_1_R and iNOS is associated with mitochondrial dysfunction, and we found both proteins to be viable targets for pharmaceutical intervention, making MRI‐1867 a strong treatment candidate. Increased oxidative and nitrosative stress fueling mitochondrial dysfunction in the lung^42^ is compatible with a role of iNOS in mitochondrial dysfunction.^53^, ^54^ Furthermore, CB_1_R‐dependent mitochondrial dysfunction of AT2 cells may also be responsible for the observed increase in lung levels of truncated oxi‐PCs, and consequently, initiation of HPSPF. Uptake of truncated oxi‐PCs by AMs is associated with conversion to foamy cells as well as M2 polarization and TGFβ‐1 secretion, both of which are features of PF initiation.^40^ Another study demonstrated that AT2 cell‐derived monocyte chemoattractant protein‐1 controls TGFβ‐1 release from AMs in pale ear mice.^34^ Here, both MRI‐1867 and rimonabant treatment almost completely attenuated bleomycin‐induced increase in TGFβ‐1 levels in BALF from pale ear mice (Figure S6D). The truncated oxi‐PCs with increased levels in PF (Figure 4) are derived from phosphatidylcholines that are abundantly produced by AT2 cells as critical surfactant components.^40^ Moreover, mitochondria play a critical role in generating surfactant phosphatidylcholines, and we found that CB_1_R blockade attenuated the increase in truncated oxi‐PC levels.^41^ Additionally, acute CB_1_R activation increased IL‐6 and TNFα in synthetic cannabinoid‐induced acute lung injury, which were completely blocked by CB_1_R antagonism. This also indicates that CB_1_R activation induces multiple proinflammatory cytokines in inflammatory lung diseases. Future studies should investigate the role of AT2‐cell CB_1_R in truncated oxi‐PC formation and regulating cytokine signaling in PF. Overall, our current findings are compatible with a pathogenic role of CB_1_R activation in HPSPF and its contribution to increased TGFβ‐1 levels (Figure S6) in pale ear mice and activated human HPSPF lung fibroblasts (Figure 7). Thus, CB_1_R is a potential therapeutic target in HPSPF.

The accelerated progression and complex pathobiology of HPSPF require a multitarget therapeutic approach. MRI‐1867 is an orally bioavailable dual‐target compound that selectively blocks peripheral CB_1_R due to its limited brain penetrance and also directly inhibits iNOS activity.^36^, ^55^ MRI‐1867 treatment also resulted in improved therapeutic efficacy relative to single target inhibitors in rodent models of liver fibrosis,^36^ obesity‐induced dyslipidemia,^56^ kidney fibrosis,^57^ and PF.^27^ Therefore, dual‐inhibition of CB_1_R and iNOS by MRI‐1867 could be a promising therapeutic strategy providing improved antifibrotic efficacy in multiple forms of organ fibrosis.^55^, ^58^

Taken together, these findings underscore important similarities between human HPSPF and pale ear mouse bleomycin‐induced HpsPF that strengthen the translational potential of this model and provides justification for developing MRI‐1867 as a candidate drug to treat patients with HPSPF.

## 4 MATERIALS AND METHODS

### 4.1 Chemicals

MRI‐1867 (the *S* enantiomer *S*‐MRI‐1867) was synthesized as described.^36^, ^59^ Rimonabant was obtained from the National Institute of Drug Abuse Drug Supply Program (Research Triangle Park, NC, USA). 1400W was from Tocris (Bristol, UK). Pharmaceutical grade bleomycin was from Hospira (Lake Forest, IL, USA). All the other chemicals were from Sigma–Aldrich (St. Louis, MO, USA).

### 4.2 Experimental drug treatment

The compounds were administered by oral gavage once daily as indicated. The vehicle was a 1:1:18 ratio of DMSO:Tween 80:Saline. Oral formulations were applied at 1 mg/ml concentrations to achieve doses of 10 mg/kg.

### 4.3 Human subject consent

Subjects with IPF, HPS, and healthy research volunteers provided written informed consent and enrolled in protocol 95‐HG‐0193 (clinicaltrials.gov NCT00001456, “Clinical and Basic Investigations into Hermansky‐Pudlak Syndrome”) or 04‐HG‐0211 (clinicaltrials.gov NCT00084305, “Procurement and Analysis of Specimens from Individuals with Pulmonary Fibrosis”), which were approved by the Institutional Review Board of the National Human Genome Research Institute. Study eligibility criteria were previously described.^60^ Human subject characteristics for BALF samples are given in Table 1.

### 4.4 PFTs for human subjects

PFT was performed as described.^61^ Briefly, forced vital capacity, total lung capacity, and diffusion capacity of carbon monoxide measurements were made in accordance with American Thoracic Society guidelines (SensorMedics, Yorba Linda, CA, USA). Values were expressed as percentages of predicted values.

### 4.5 BALF and lung tissue procurement for human subjects

BALF was isolated and lung tissue fixed in 10% formalin. Both were procured as described.^62^, ^63^

### 4.6 Animals

All animal procedures were conducted in accordance with the rules and regulations of the Institutional Animal Care and Use Committee of the National Institutes of Alcohol Abuse and Alcoholism (NIAAA) and the National Human Genome Research Institute (NHGRI), under the protocols of LPS‐GK1 and G‐14‐3, respectively. Thirteen‐week‐old male C57BL/6J mice were obtained from The Jackson Laboratory (Bar Harbor, ME, USA). Pale ear mice were on a C57Bl/6J genetic background. Mice were housed individually under a 12‐h light/dark cycle and fed a standard diet, *ad libitum* (Teklad NIH‐31; Envigo, Huntingdon, UK).

### 4.7 Oropharyngeal aspiration of bleomycin

We generated a bleomycin‐induced PF model by delivering bleomycin via oropharyngeal aspiration as previously described.^39^ Briefly, bleomycin (Hospira) is delivered to mice anesthetized with ketamine/xylazine through the oropharynx at 0.3 U/kg using a sterile 100 μl pipette during inspiration at a volume of 100 μl/50 g body weight. Sterile saline was used as vehicle and applied to the control groups. The animals are then allowed to recover from the anesthesia.

### 4.8 Subcutaneous osmotic delivery of bleomycin

We generated a bleomycin‐induced PF model by delivering bleomycin (60 U/kg/7 days) via subcutaneous osmotic delivery^39^ as previously described.

### 4.9 BALF harvesting from mice

BALF was collected from anesthetized mice by lavaging lungs three times with 1 ml Hanks’ balanced salt solution without calcium and magnesium (HBS‐) (Sigma Aldrich). Supernatant was collected as BALF for further analysis and 500 μl of BALF was subjected to endocannabinoid measurements.

### 4.10 Lung function measurements in pale ear mice

Respiratory system mechanics measurements were performed using the FlexiVent FX system (SCIREQ Inc., Montreal, Canada), which is equipped with an FX1 module and negative pressure forced expiration extension for mice. FlexiWare v7.2 software was used to operate the system. Forced oscillation techniques and forced expiration measurements were conducted as described previously.^64^, ^65^ Lung function measurements were performed at the end of the study as a terminal procedure. Mice were anesthetized by intraperitoneal (IP) injection of Ketamine/Xylazine, then an 18‐gauge metal cannula was inserted into the trachea by small incision. Pancuronium was then administered by IP injection (0.8 mg/kg) to induce paralysis before connecting mice to FlexiVent and starting ventilation. Pressure‐volume (PV) curve, airway resistance, tissue damping (G), tissue elastance (H), and forced expiratory volume per 0.1 s (FEV 0.1) parameters were measured. Mouse tissue was collected after performing lung function tests.

### 4.11 Real‐time PCR analyses

RNA extraction was performed using RNeasy Mini Kits from Qiagen (Valencia, CA). One microgram of total RNA was reverse transcribed to cDNA using Bio‐Rad iScript cDNA synthesis kit (Hercules, CA). Expression of the target gene was quantified with gene‐specific primers and PowerSYBRGreen master mix using a StepOnePlus Real‐Time PCR instrument from Applied Biosystems. Predesigned mouse *Tbp* (QT00198443, *Col1a* (QT00162204)*, Nos2* (QT00100275), *Cnr1* (QT00115395), *Ppargc1a* (QT00156303), *Pink1* (QT00111349), and *IL11* (QT00122122, QT00074088) primers were purchased from Qiagen. The house‐keeping gene TATA‐Box Binding Protein (*Tbp*) was used as the loading control. Gene expression values were calculated based on the ∆∆Ct method.

### 4.12 Cytokine measurements

Level of IL‐11 in fibroblast culture media was measured using the IL‐11 human ELISA Kit (Abcam). Level of TGF‐β1 in BALF was measured using TGF‐β1 quantikine ELISA kit (R&D systems).

### 4.13 Endocannabinoid measurement

The tissue levels of endocannabinoids were measured by stable isotope dilution liquid chromatography/tandem mass spectrometry (LC‐MS/MS) as described.^36^

### 4.14 Tissue levels of drugs

MRI‐1867 levels were measured by LC‐MS/MS as described.^36^

### 4.15 Hyp measurement

The degree of lung fibrosis was quantified biochemically by measuring Hyp content of lung extracts using LC‐MS/MS as described.^39^

### 4.16 Fluorescent *in situ* hybridization

Fluorescent *in situ* hybridization (FISH) was performed using the RNAscope® Multiplex Fluorescent Kit v2 (Advanced Cell Diagnostics [ACD], Newark, CA, USA, Cat. 323110) as previously described^39^ using formalin fixed paraffin embedded (FFPE) lung tissues from mice. Images were collected using the Zeiss LSM700 confocal microscope. The probes used from ACD were *Col1a1* in channel 2 (Cat. 319371‐C2), *Sftpc* in channel 2 (Cat. 314101‐C2), *Cd68* in channel 2 (Cat. 316611‐C2), *Cnr1* in channel 1 (Cat. 420721), *Nos2* in channel 1 (Cat. 319131), and *IL11* in channel 1 (Cat. 552461). The fluorophores used to visualize the RNAscope® ISH probes were cyanine 3 and 5 from the TSA Cyanine 3 & 5, TMR, Fluoresein Evaluation Kit (Perkin Elmer, Waltham, MA, USA; Cat. NEL760001KT). Cyanine 3 and 5 were diluted in RNAscope® LS Multiplex TSA Buffer (ACD, Cat. 322810). The fluorophore dilutions for each RNAscope® ISH probe were *Cd68*‐Cyanine 5 (1:750), *Sftpc*‐Cyanine 5 (1:15000), and *Col1a1*‐Cyanine 5 (1:7500). The positive control probes for determining lung tissue RNA quality were *Polr2a* in channel 1 and *Ubc* in channel 3 from the RNAscope® 3‐Plex Positive Control Probes (ACD, Cat. 320881). Fluorophore dilutions for positive control probes were *Polr2a*‐Cyanine 3 (1:750) and *Ubc*‐Cyanine 5 (1:750). The negative control probe for determining nonspecific background staining was bacterial gene *dapB* in channels 1 and 3 from the RNAscope® 3‐Plex Negative Control Probe (ACD, Cat. 320871). The fluorophore concentrations for the negative control probe were *dapB*‐channel 1‐Cyanine 3 (1:750) and *dapB*‐channel 3 Cyanine 5 (1:750). All washes, AMP steps, and blocking steps were performed according to the RNAscope® Multiplex Fluorescent Reagent Kit v2 User Manual.

### 4.17 Histology and immunohistochemistry

The right lung was used for histology for mice. During lung harvesting, first the left bronchial arm was ligated, and the left lung removed for biochemical analyses. The remaining right lung was then inflated by 1.5 ml of 10% neutralized formalin solution introduced into the trachea using a syringe. The right bronchial arm was then ligated to keep the lung inflated before its removal. The right lung was then fixed in 10% neutralized formalin solution, embedded in paraffin and sectioned (4 μm) onto glass slides. Immunohistochemistry was performed as described^36^ by using primary antibodies against iNOS (Abcam 15323), CB_1_R (anti‐mouse L15).^66^ Immunostaining intensity was quantified by using IMAGEJ software (NIH Public Domain) by a person blind to the sample ID. Quantification of images from one sample was averaged for actual data presentation for each sample *n* = 6 per group.

### 4.18 Masson's trichrome staining

Histological staining was performed using Masson's Trichrome Kit (Thermo Fisher Scientific) with a slight optimization of the supplier's microwave staining protocol. Four micrometers tissue sections were stained with the following time adjustments: DI water rinse adjusted to 5 min after Bouin's Fluid; Weigert's Iron Hematoxylin stain adjusted to 3 min; Biebrich Scarlet‐Acid Fuchsin solution adjusted to 2 min; and Aniline Blue Solution adjusted to 25 min. All other steps were performed as instructed.

### 4.19 Ashcroft scoring

Images were taken at 200X magnification from at least eight randomly selected areas per lung tissue slides. Three readers scored the same fields independently (0 = no fibrosis; 8 = severe fibrosis) and were blinded to study group.

### 4.20 Survival analysis for mice

Survival curves were plotted by using GraphPad Prism 8 software. Survival was analyzed by using Log‐rank (Mantel–Cox) test for assessing statistical difference between animal groups.

### 4.21 Primary lung fibroblasts isolation and treatments

Explanted lung tissue samples were obtained from patients with genetically confirmed HPS‐1 who underwent clinically indicated lung transplantation for HPSPF. Lung fibroblasts were cultured in growth media containing 10% Mesenchymal Cell Growth Supplement, 2% L‐Glutamine, and 0.1% Gentamicin Sulfate in Lonza's Mesenchymal Stem Cell Basal Medium (Lonza, Switzerland). Control lung fibroblasts were purchased from Lonza and maintained in the same growth media. When the fibroblasts reached 80–90% confluency, cells were incubated with either 1 μM MRI‐1867, 1 μM Rimonabant, 1 μM 1400W, or DMSO for vehicle and fibrosis‐only control. One hour later, the cells were stimulated with 10 ng/ml TGFβ‐1 (240‐B‐010, R&D Systems) diluted in 4 mM HCL in 1 mg/ml BSA (R&D Systems), while the vehicle and fibrosis‐only control were incubated with equal volume of diluent. Twenty‐four hours later, culture supernatant was collected and rapidly frozen for cytokine and endocannabinoid measurement. Cells were washed three times with PBS, trypsinized, washed, pelleted, and frozen. RNA was collected using Maxwell® RSC simplyRNA Cells Kit (AS1390) on a Maxwell® RSC Instrument (AS4500, Promega). RNA quality and quantity were measured using QuantiFluor® RNA System (E3310) on a Quantus™ Fluorometer (E6150, Promega). One microgram of RNA was transcribed to cDNA as above.

### 4.22 Statistical analysis

Statistical analysis was performed by unpaired two‐tailed Student's *t* test or one‐way ANOVA, as appropriate and indicated in figure legends. *p *< 0.05 was considered significant.

## AUTHOR CONTRIBUTIONS

R.C. designed the study, planned experiments, performed mass spectrometry experiments, analyzed data, and drafted the manuscript. T.J., J.K.P., N.J.C., J.A., L.J., and M.X.G.Z. performed histology, immunohistochemistry, RNAscope, and acquired images. J.K.P., N.J.C., C.N.Z., and J.A. performed qPCR and edited the manuscript. K.M. contributed the CB_1_R antibody and edited the manuscript. R.C., J.K.P., C.N.Z., N.J.C., S.P.B, and T.Y. contributed to *in vivo* animal experiments. M.R.I., J.H., and A.A. synthesized and chemically analyzed MRI‐1867. M.C.V.M. contributed to design animal experiments and performed human fibroblast experiments. B.R.G. and K.J.O. obtained clinical samples and performed pulmonary function tests. B.R.G., T.Y., and M.C.V.M. performed Ashcroft scoring. R.C., B.R.G., M.C.V.M., W.A.G., and G.K. interpreted data and participated in manuscript preparation. All authors reviewed the manuscript and agreed with the final version.

## CONFLICTS OF INTEREST

R. Cinar, M.R. Iyer, and G. Kunos are listed as coinventors on a US patent covering MRI‐1867 and related compounds (patent no. US 9,765,031 B2).

## Supporting information

Supporting Information

## References

[1] Huizing M , Helip‐Wooley A , Westbroek W , Gunay‐Aygun M , Gahl WA . Disorders of lysosome‐related organelle biogenesis: clinical and molecular genetics. Annu Rev Genomics Hum Genet. 2008;9:359‐386. 18544035 10.1146/annurev.genom.9.081307.164303 PMC2755194

[2] Sanchez‐Guiu I , Torregrosa JM , Velasco F , et al. Hermansky–Pudlak syndrome. Overview of clinical and molecular features and case report of a new HPS‐1 variant. Hamostaseologie. 2014;34(4):301‐309. 25117010 10.5482/HAMO-14-06-0024

[3] Yokoyama T , Gochuico BR . Hermansky–Pudlak syndrome pulmonary fibrosis: a rare inherited interstitial lung disease. European Respiratory Review. 2021;30(159):200193. 10.1183/16000617.0193-2020. 33536261 PMC9488956

[4] Brantly M , Avila NA , Shotelersuk V , Lucero C , Huizing M , Gahl WA . Pulmonary function and high‐resolution CT findings in patients with an inherited form of pulmonary fibrosis, Hermansky–Pudlak syndrome, due to mutations in HPS‐1. Chest. 2000;117(1):129‐136. 10631210 10.1378/chest.117.1.129

[5] Gochuico BR , Huizing M , Golas GA , et al. Interstitial lung disease and pulmonary fibrosis in Hermansky–Pudlak syndrome type 2, an adaptor protein‐3 complex disease. Mol Med. 2012;18:56‐64. 22009278 10.2119/molmed.2011.00198 PMC3269640

[6] White DA , Smith GJ , Cooper JA Jr , Glickstein M , Rankin JA . Hermansky–Pudlak syndrome and interstitial lung disease: report of a case with lavage findings. Am Rev Respir Dis. 1984;130(1):138‐141. 6742598 10.1164/arrd.1984.130.1.138

[7] Nakatani Y , Nakamura N , Sano J , et al. Interstitial pneumonia in Hermansky–Pudlak syndrome: significance of florid foamy swelling/degeneration (giant lamellar body degeneration) of type‐2 pneumocytes. Virchows Arch. 2000;437(3):304‐313. 11037352 10.1007/s004280000241

[8] O'Brien K , Troendle J , Gochuico BR , et al. Pirfenidone for the treatment of Hermansky–Pudlak syndrome pulmonary fibrosis. Mol Genet Metab. 2011;103(2):128‐134. 21420888 10.1016/j.ymgme.2011.02.003 PMC3656407

[9] Gahl WA , Brantly M , Troendle J , et al. Effect of pirfenidone on the pulmonary fibrosis of Hermansky–Pudlak syndrome. Mol Genet Metab. 2002;76(3):234‐242. 12126938 10.1016/s1096-7192(02)00044-6

[10] Rouhani FN , Brantly ML , Markello TC , et al. Alveolar macrophage dysregulation in Hermansky–Pudlak syndrome type 1. Am J Respir Crit Care Med. 2009;180(11):1114‐1121. 19729668 10.1164/rccm.200901-0023OC PMC2784416

[11] El‐Chemaly S , Young LR . Hermansky–Pudlak syndrome. Clin Chest Med. 2016;37(3):505‐511. 27514596 10.1016/j.ccm.2016.04.012 PMC4987498

[12] Atochina‐Vasserman EN , Bates SR , Zhang P , et al. Early alveolar epithelial dysfunction promotes lung inflammation in a mouse model of Hermansky–Pudlak syndrome. Am J Respir Crit Care Med. 2011;184(4):449‐458. 21616998 10.1164/rccm.201011-1882OC PMC3175543

[13] Young LR , Borchers MT , Allen HL , Gibbons RS , McCormack FX . Lung‐restricted macrophage activation in the pearl mouse model of Hermansky–Pudlak syndrome. J Immunol. 2006;176(7):4361‐4368. 16547274 10.4049/jimmunol.176.7.4361 PMC3783655

[14] Kalayarasan S , Sriram N , Sudhandiran G . Diallyl sulfide attenuates bleomycin‐induced pulmonary fibrosis: critical role of iNOS, NF‐kappaB, TNF‐alpha and IL‐1beta. Life Sci. 2008;82(23‐24):1142‐1153. 18462759 10.1016/j.lfs.2008.03.018

[15] Naura AS , Zerfaoui M , Kim H , et al. Requirement for inducible nitric oxide synthase in chronic allergen exposure‐induced pulmonary fibrosis but not inflammation. J Immunol. 2010;185(5):3076‐3085. 20668217 10.4049/jimmunol.0904214 PMC3077076

[16] Pullamsetti SS , Savai R , Dumitrascu R , et al. The role of dimethylarginine dimethylaminohydrolase in idiopathic pulmonary fibrosis. Sci Transl Med. 2011;3(87):87ra53. 10.1126/scitranslmed.3001725 21677199

[17] Romanska HM , Polak JM , Coleman RA , et al. iNOS gene upregulation is associated with the early proliferative response of human lung fibroblasts to cytokine stimulation. J Pathol. 2002;197(3):372‐379. 12115884 10.1002/path.1116

[18] Romanska HM , Ikonen TS , Bishop AE , Morris RE , Polak JM . Up‐regulation of inducible nitric oxide synthase in fibroblasts parallels the onset and progression of fibrosis in an experimental model of post‐transplant obliterative airway disease. J Pathol. 2000;191(1):71‐77. 10767722 10.1002/(SICI)1096-9896(200005)191:1<71::AID-PATH560>3.0.CO;2-I

[19] Hsu YC , Wang LF , Chien YW . Nitric oxide in the pathogenesis of diffuse pulmonary fibrosis. Free Radic Biol Med. 2007;42(5):599‐607. 17291983 10.1016/j.freeradbiomed.2006.11.031

[20] Teixeira‐Clerc F , Julien B , Grenard P , et al. CB1 cannabinoid receptor antagonism: a new strategy for the treatment of liver fibrosis. Nat Med. 2006;12(6):671‐676. 16715087 10.1038/nm1421

[21] Trebicka J , Racz I , Siegmund SV , et al. Role of cannabinoid receptors in alcoholic hepatic injury: steatosis and fibrogenesis are increased in CB2 receptor‐deficient mice and decreased in CB1 receptor knockouts. Liver Int. 2011;31(6):860‐870. 21645218 10.1111/j.1478-3231.2011.02496.x

[22] Reichenbach V , Ros J , Fernandez‐Varo G , et al. Prevention of fibrosis progression in CCl4‐treated rats: role of the hepatic endocannabinoid and apelin systems. J Pharmacol Exp Ther. 2012;340(3):629‐637. 22160265 10.1124/jpet.111.188078 PMC11047215

[23] Lin CL , Hsu YC , Lee PH , et al. Cannabinoid receptor 1 disturbance of PPARgamma2 augments hyperglycemia induction of mesangial inflammation and fibrosis in renal glomeruli. J Mol Med. 2014;92(7):779‐792. 24722948 10.1007/s00109-014-1125-6

[24] Slavic S , Lauer D , Sommerfeld M , et al. Cannabinoid receptor 1 inhibition improves cardiac function and remodelling after myocardial infarction and in experimental metabolic syndrome. J Mol Med. 2013;91(7):811‐823. 23636507 10.1007/s00109-013-1034-0

[25] Lazzerini PE , Natale M , Gianchecchi E , et al. Adenosine A2A receptor activation stimulates collagen production in sclerodermic dermal fibroblasts either directly and through a cross‐talk with the cannabinoid system. J Mol Med. 2012;90(3):331‐342. 22033526 10.1007/s00109-011-0824-5

[26] Bronova I , Smith B , Aydogan B , et al. Protection from Radiation‐Induced Pulmonary Fibrosis by Peripheral Targeting of Cannabinoid Receptor‐1. Am J Respir Cell Mol Biol. 2015;53(4):555–562. 26426981 10.1165/rcmb.2014-0331OC PMC4742897

[27] Cinar R , Gochuico BR , Iyer MR , et al. Cannabinoid CB1 receptor overactivity contributes to the pathogenesis of idiopathic pulmonary fibrosis. JCI Insight. 2017;2(8):e92281 10.1172/jci.insight.92281 PMC5396529 28422760

[28] Han X , Sun YJ , Scott S , Bleich D . Tissue inhibitor of metalloproteinase‐1 prevents cytokine‐mediated dysfunction and cytotoxicity in pancreatic islets and beta‐cells. Diabetes. 2001;50(5):1047‐1055. 11334407 10.2337/diabetes.50.5.1047

[29] Liu J , Zhou L , Xiong K , et al. Hepatic cannabinoid receptor‐1 mediates diet‐induced insulin resistance via inhibition of insulin signaling and clearance in mice. Research Support, N.I.H., Intramural. Gastroenterology. 2012;142(5):1218‐1228 e1. 22307032 10.1053/j.gastro.2012.01.032 PMC3482511

[30] Soliman E , Henderson KL , Danell AS , Van Dross R . Arachidonoyl‐ethanolamide activates endoplasmic reticulum stress‐apoptosis in tumorigenic keratinocytes: role of cyclooxygenase‐2 and novel J‐series prostamides. Mol Carcinog. 2016.55(2):117–130. 25557612 10.1002/mc.22257

[31] Salazar M , Carracedo A , Salanueva IJ , et al. Cannabinoid action induces autophagy‐mediated cell death through stimulation of ER stress in human glioma cells. J Clin Invest. 2009;119(5):1359‐1372. 19425170 10.1172/JCI37948 PMC2673842

[32] Tanjore H , Lawson WE , Blackwell TS . Endoplasmic reticulum stress as a pro‐fibrotic stimulus. Biochim Biophys Acta. 2013;1832(7):940‐947. 23201247 10.1016/j.bbadis.2012.11.011 PMC3641173

[33] Torres‐Gonzalez E , Bueno M , Tanaka A , et al. Role of endoplasmic reticulum stress in age‐related susceptibility to lung fibrosis. Am J Respir Cell Mol Biol. 2012;46(6):748‐756. 22227563 10.1165/rcmb.2011-0224OC PMC3380287

[34] Young LR , Gulleman PM , Short CW , et al. Epithelial–macrophage interactions determine pulmonary fibrosis susceptibility in Hermansky–Pudlak syndrome. JCI Insight. 2016;1(17):e88947. 27777976 10.1172/jci.insight.88947 PMC5070955

[35] Young LR , Pasula R , Gulleman PM , Deutsch GH , McCormack FX . Susceptibility of Hermansky–Pudlak mice to bleomycin‐induced type II cell apoptosis and fibrosis. Am J Respir Cell Mol Biol. 2007;37(1):67‐74. 17363777 10.1165/rcmb.2006-0469OC PMC1899346

[36] Cinar R , Iyer MR , Liu Z , et al. Hybrid inhibitor of peripheral cannabinoid‐1 receptors and inducible nitric oxide synthase mitigates liver fibrosis. JCI Insight. 2016;1(11):e87336. 10.1172/jci.insight.87336 PMC4979564 27525312

[37] Gardner JM , Wildenberg SC , Keiper NM , et al. The mouse pale ear (ep) mutation is the homologue of human Hermansky–Pudlak syndrome. Proc Natl Acad Sci U S A. 1997;94(17):9238‐9243. 9256466 10.1073/pnas.94.17.9238 PMC23134

[38] Lyerla TA , Rusiniak ME , Borchers M , et al. Aberrant lung structure, composition, and function in a murine model of Hermansky–Pudlak syndrome. Am J Physiol Lung Cell Mol Physiol. 2003;285(3):L643‐L653. 12777251 10.1152/ajplung.00024.2003

[39] Park JK , Coffey NJ , Bodine SP , et al. Bleomycin induces drug efflux in lungs. A pitfall for pharmacological studies of pulmonary fibrosis. Am J Respir Cell Mol Biol. 2020;62(2):178‐190. 31419911 10.1165/rcmb.2018-0147OC PMC6993545

[40] Romero F , Shah D , Duong M , et al. A pneumocyte‐macrophage paracrine lipid axis drives the lung toward fibrosis. Am J Respir Cell Mol Biol. 2015;53(1):74‐86. 25409201 10.1165/rcmb.2014-0343OC PMC4566113

[41] Zawatsky CN , Abdalla J , Cinar R . Synthetic cannabinoids induce acute lung inflammation via cannabinoid receptor 1 activation. ERJ Open Res. 2020;6(3):121. 10.1183/23120541.00121-2020 PMC7430153 32832534

[42] Zank DC , Bueno M , Mora AL , Rojas M . Idiopathic pulmonary fibrosis: aging, mitochondrial dysfunction, and cellular bioenergetics. Front Med (Lausanne). 2018;5:10. 29459894 10.3389/fmed.2018.00010 PMC5807592

[43] Tedesco L , Valerio A , Dossena M , et al. Cannabinoid receptor stimulation impairs mitochondrial biogenesis in mouse white adipose tissue, muscle, and liver: the role of eNOS, p38 MAPK, and AMPK pathways. Diabetes. 2010;59(11):2826‐2836. 20739683 10.2337/db09-1881 PMC2963541

[44] Godlewski G , Cinar R , Coffey NJ , et al. Targeting peripheral CB1 receptors reduces ethanol intake via a gut–brain axis. Cell Metab. 2019;29:1320‐1333. 31105045 10.1016/j.cmet.2019.04.012 PMC6551287

[45] Drori A , Permyakova A , Hadar R , Udi S , Nemirovski A , Tam J . Cannabinoid‐1 receptor regulates mitochondrial dynamics and function in renal proximal tubular cells. Diabetes Obes Metab. 2019;21(1):146‐159. 30091204 10.1111/dom.13497 PMC6586028

[46] Bueno M , Lai YC , Romero Y , et al. PINK1 deficiency impairs mitochondrial homeostasis and promotes lung fibrosis. J Clin Invest. 2015;125(2):521‐538. 25562319 10.1172/JCI74942 PMC4319413

[47] Ng B , Dong J , D'Agostino G , et al. Interleukin‐11 is a therapeutic target in idiopathic pulmonary fibrosis. Sci Transl Med. 2019;11(511).eaaw1237 31554736 10.1126/scitranslmed.aaw1237

[48] Strikoudis A , Cieslak A , Loffredo L , et al. Modeling of fibrotic lung disease using 3D organoids derived from human pluripotent stem cells. Cell Rep. 2019;27(12):3709‐3723. 31216486 10.1016/j.celrep.2019.05.077 PMC6594401

[49] Jenkins RG , Moore BB , Chambers RC , et al. An Official American Thoracic Society Workshop Report: use of animal models for the preclinical assessment of potential therapies for pulmonary fibrosis. Am J Respir Cell Mol Biol. 2017;56(5):667‐679. 28459387 10.1165/rcmb.2017-0096ST PMC5800895

[50] Parra ER , Aguiar Junior AC , Silva LO , Souza HS , Espinoza JD , Capelozzi VL . Morphometric evaluation of nitric oxide synthase isoforms and their cytokine regulators predict pulmonary dysfunction and survival in systemic sclerosis. Braz J Med Biol Res. 2013;46(10):881‐891. 24141615 10.1590/1414-431X20133061 PMC3854315

[51] Genovese T , Cuzzocrea S , Di Paola R , et al. Inhibition or knock out of inducible nitric oxide synthase result in resistance to bleomycin‐induced lung injury. Respir Res. 2005;6:58. 15955252 10.1186/1465-9921-6-58 PMC1177992

[52] Cui H , Xie N , Banerjee S , Ge J , Guo S , Liu G . Impairment of fatty acid oxidation in alveolar epithelial cells mediates acute lung injury. Am J Respir Cell Mol Biol. 2019;60(2):167‐178. 30183330 10.1165/rcmb.2018-0152OC PMC6376408

[53] Jeon MJ , Leem J , Ko MS , et al. Mitochondrial dysfunction and activation of iNOS are responsible for the palmitate‐induced decrease in adiponectin synthesis in 3T3L1 adipocytes. Exp Mol Med. 2012;44(9):562‐570. 22809900 10.3858/emm.2012.44.9.064 PMC3465750

[54] Brown GC . Regulation of mitochondrial respiration by nitric oxide inhibition of cytochrome c oxidase. Biochim Biophys Acta. 2001;1504(1):46‐57. 11239484 10.1016/s0005-2728(00)00238-3

[55] Cinar R , Iyer MR , Kunos G . The therapeutic potential of second and third generation CB1R antagonists. Pharmacol Ther. 2020;208:107477. 31926199 10.1016/j.pharmthera.2020.107477 PMC8605822

[56] Roger C , Buch C , Muller T , et al. Simultaneous inhibition of peripheral CB1R and iNOS mitigates obesity‐related dyslipidemia through distinct mechanisms. Diabetes. 2020.69(10):2120–2132. 32680936 10.2337/db20-0078 PMC7506827

[57] Udi S , Hinden L , Ahmad M , et al. Dual inhibition of cannabinoid CB1 receptor and inducible NOS attenuates obesity‐induced chronic kidney disease. Br J Pharmacol. 2020;177(1):110‐127. 31454063 10.1111/bph.14849 PMC6976880

[58] Cinar Resat , Iyer Malliga R. , Kunos George . Dual inhibition of CB1 receptors and iNOS, as a potential novel approach to the pharmacological management of acute and long COVID‐19. British Journal of Pharmacology. 2021; 10.1111/bph.15461. PMC8251289 33769552

[59] Alimardanov A , Huang J . A scalable synthesis of dual‐target inhibitor of cannabinoid‐1 receptor and inducible nitric oxide synthase. Patent application PCT/US20/030624. 2020.

[60] El‐Chemaly S , Pacheco‐Rodriguez G , Malide D , et al. Nuclear localization of vascular endothelial growth factor‐D and regulation of c‐Myc‐dependent transcripts in human lung fibroblasts. Am J Respir Cell Mol Biol. 2014;51(1):34‐42. 24450584 10.1165/rcmb.2013-0417OC PMC4091860

[61] Gochuico BR , Avila NA , Chow CK , et al. Progressive preclinical interstitial lung disease in rheumatoid arthritis. Arch Intern Med. 2008;168(2):159‐166. 18227362 10.1001/archinternmed.2007.59

[62] Ren P , Rosas IO , Macdonald SD , Wu HP , Billings EM , Gochuico BR . Impairment of alveolar macrophage transcription in idiopathic pulmonary fibrosis. Am J Respir Crit Care Med. 2007;175(11):1151‐1157. 17332483 10.1164/rccm.200607-958OC PMC1899274

[63] Cullinane AR , Yeager C , Dorward H , et al. Dysregulation of galectin‐3. Implications for Hermansky–Pudlak syndrome pulmonary fibrosis. Am J Respir Cell Mol Biol. 2014;50(3):605‐613. 24134621 10.1165/rcmb.2013-0025OC PMC4068929

[64] McGovern TK , Robichaud A , Fereydoonzad L , Schuessler TF , Martin JG . Evaluation of Respiratory System Mechanics in Mice using the Forced Oscillation Technique. Journal of Visualized Experiments. 2013;(75):10.3791/50172. PMC3684007 23711876

[65] Devos FC , Maaske A , Robichaud A , et al. Forced expiration measurements in mouse models of obstructive and restrictive lung diseases. Respir Res. 2017;18(1):123. 28629359 10.1186/s12931-017-0610-1 PMC5477381

[66] Bodor AL , Katona I , Nyiri G , et al. Endocannabinoid signaling in rat somatosensory cortex: laminar differences and involvement of specific interneuron types. J Neurosci. 2005;25(29):6845‐6856. 16033894 10.1523/JNEUROSCI.0442-05.2005 PMC6725346

