# Supplementary material for: CB1R and iNOS are distinct players promoting pulmonary fibrosis in Hermansky–Pudlak syndrome
Source: Clin Transl Med. 2021 Jul 4;11(7):e471. doi: 10.1002/ctm2.471 (PMC8255071; doi:10.1002/ctm2.471)

Supporting Information

CB_1R and iNOS are distinct players promoting pulmonary fibrosis in Hermansky-Pudlak syndrome

Resat Cinar[1]*, Joshua K. Park[1], Charles N. Zawatsky[1], Nathan J. Coffey[1], Steven P. Bodine[2], Jasmina Abdalla[1], Tadafumi Yokoyama[2†], Tony Jourdan[1‡], Lindsey Jay[1], Mei Xing G. Zuo[1], Kevin J. O'Brien[2], Junfeng Huang[3], Ken Mackie[4], Asaf Alimardanov[3], Malliga R. Iyer[1], William A. Gahl[2,5], George Kunos[1], Bernadette R. Gochuico[2], May Christine V. Malicdan[2,5]

[1]Laboratory of Physiologic Studies, National Institute on Alcohol Abuse and Alcoholism, National Institutes of Health, Rockville, MD, 20852, USA.

[2]Section of Human Biochemical Genetics, Medical Genetics Branch, National Human Genome Research Institute, National Institutes of Health, Bethesda, MD, 20892, USA.

[3]Therapeutics Development Branch, Division of Preclinical Innovation, National Center for Advancing Translational Sciences, National Institutes of Health, Rockville, MD

[4]Department of Psychological and Brain Sciences, Indiana University, Bloomington, IN

[5]NIH Undiagnosed Diseases Program and Office of the Clinical Director, National Human Genome Research Institute, National Institutes of Health, Bethesda, MD 20892, USA.

*To whom correspondence should be addressed: Resat Cinar, BPharm, PhD (resat.cinar@nih.gov)

† Department of Pediatrics, Kanazawa University, Kanazawa Japan

‡ INSERM Lipids, Nutrition, Cancer UMR1231, University of Burgundy and Franche-Comté, Dijon, France

Running Title: Dual targeting of CB_1R and iNOS in HPSPF

Supplemental Figure 1

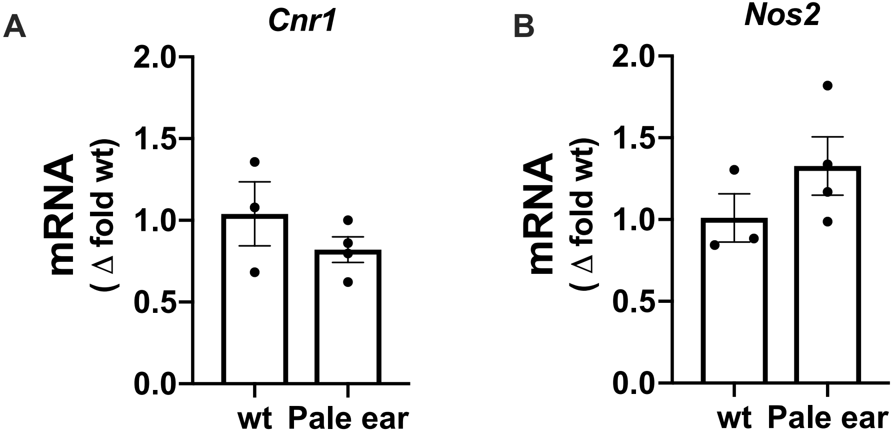

Supplemental Figure 2

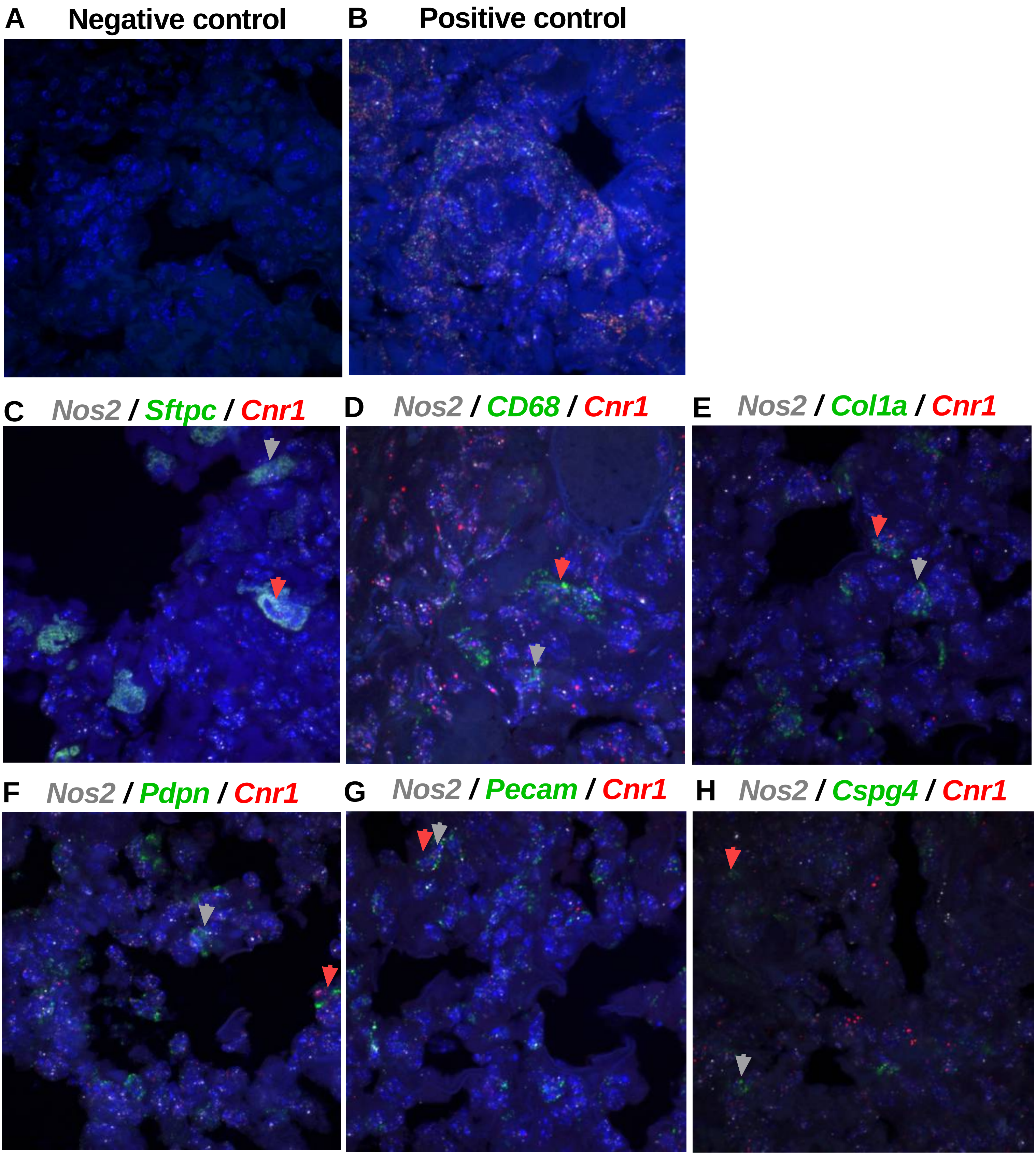

Supplemental Figure 3

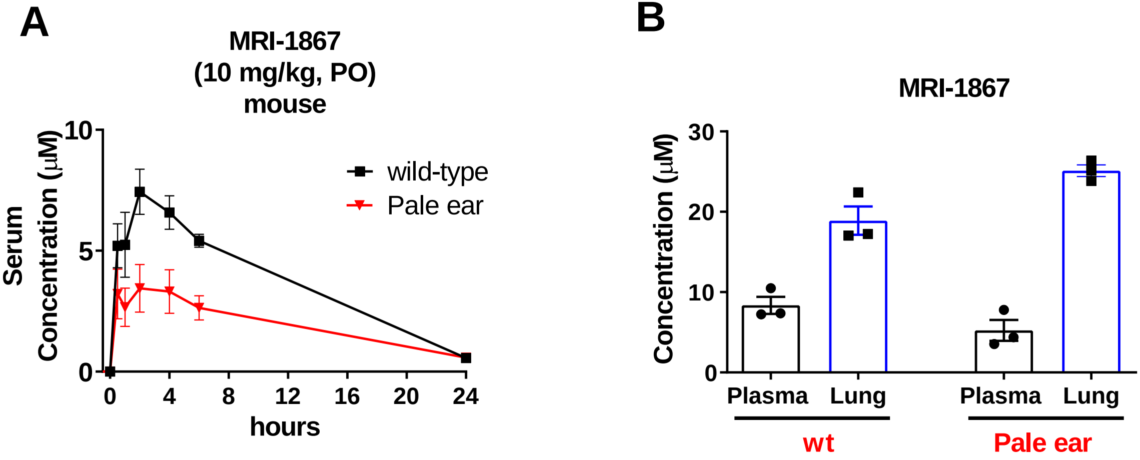

Supplemental Figure 4

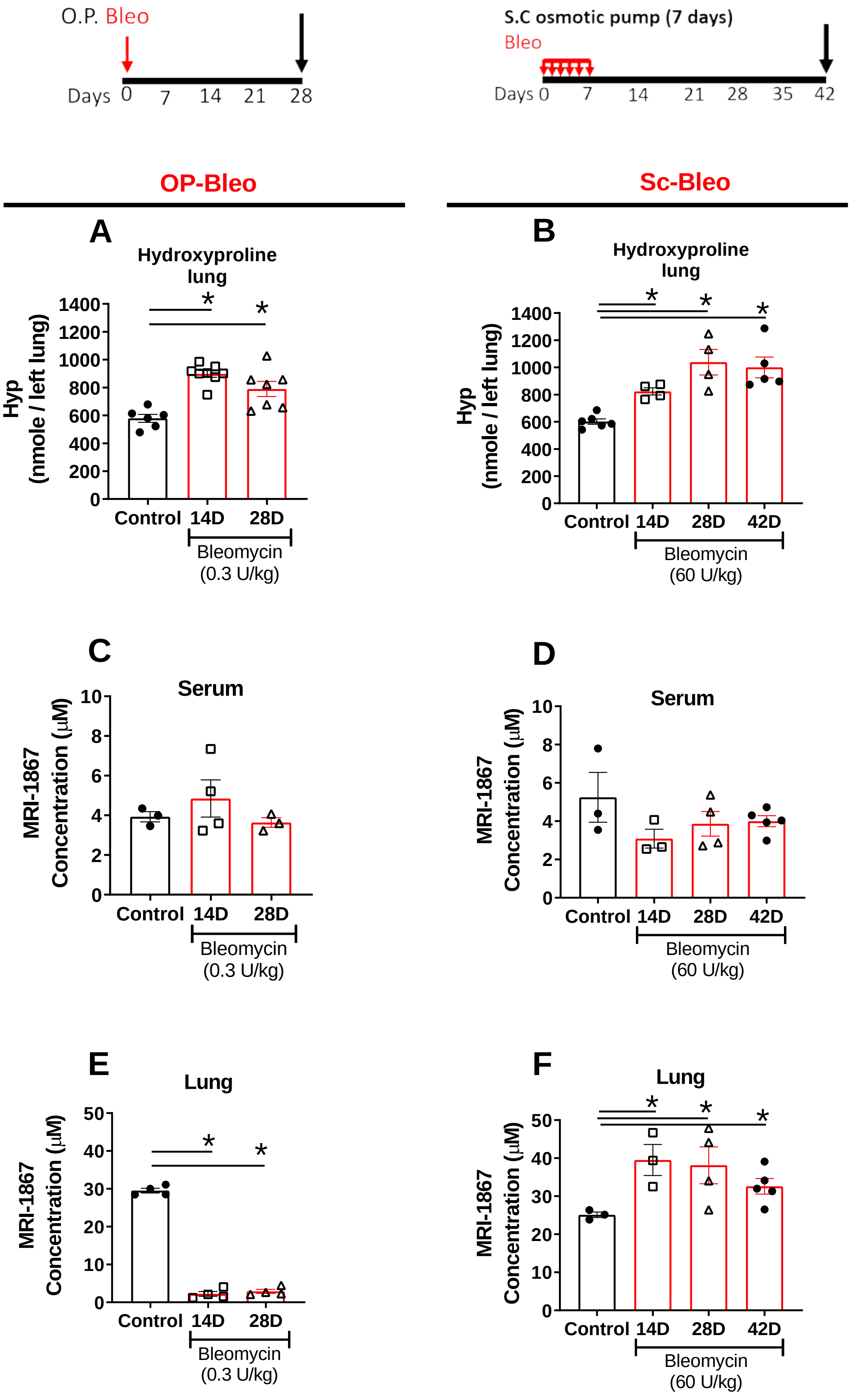

Supplemental Figure 5

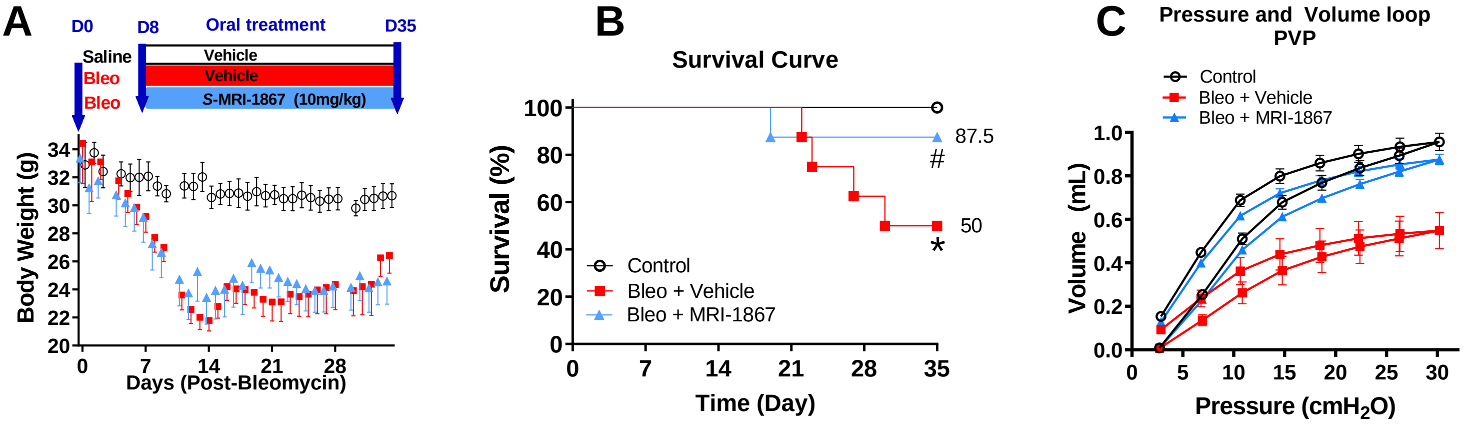

Supplemental Figure 6

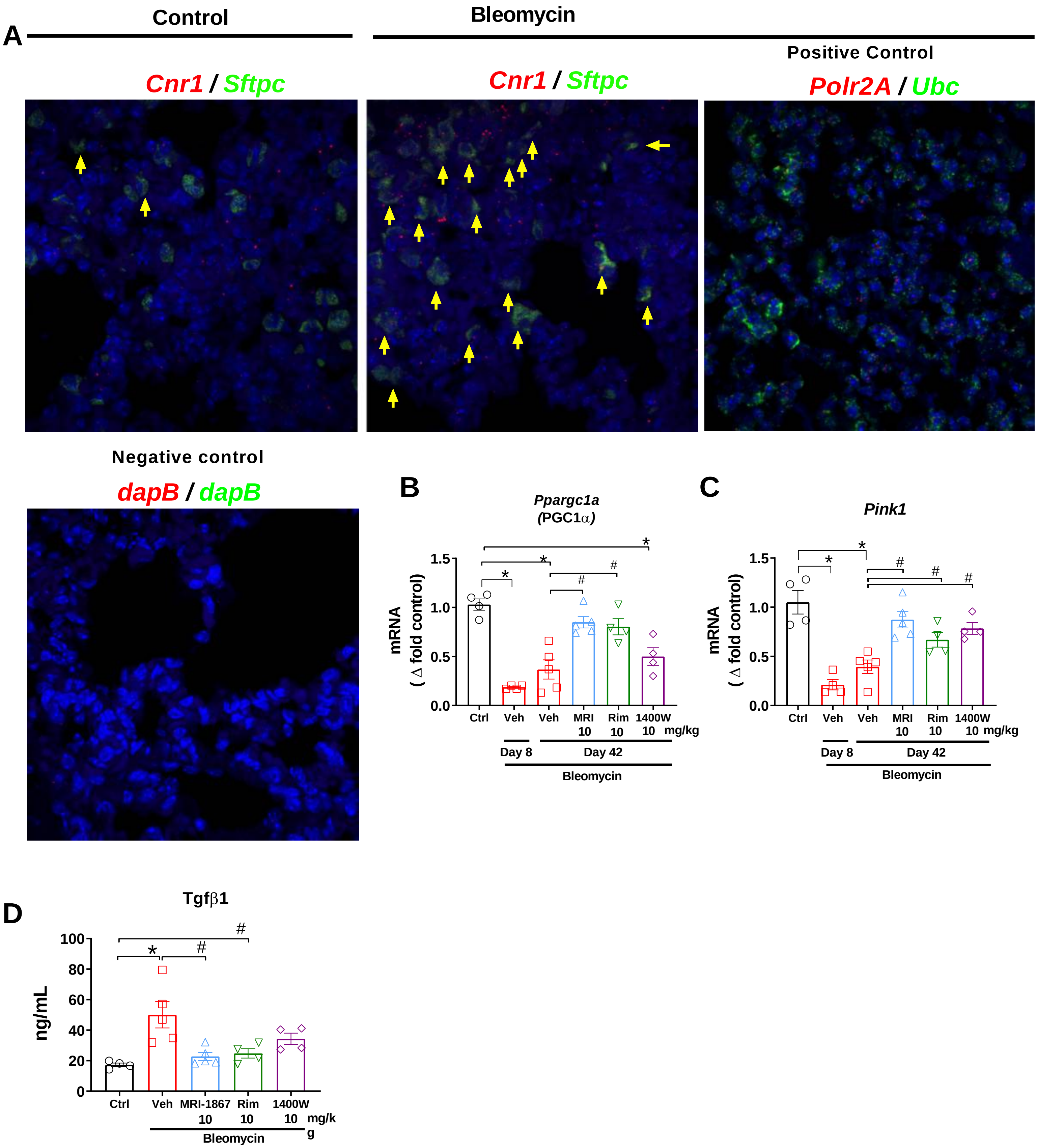

Supplement: Supplementary file 1 — Supporting Information [file CTM2-11-e471-s001.pdf]
